# Supplementary material for: Ligand-mediate exciton allocation enables efficient cluster-based white light-emitting diodes via single and heavy doping
Source: Nat Commun. 2024 Jun 12;15:4997. doi: 10.1038/s41467-024-49394-8 (PMC11169358; doi:10.1038/s41467-024-49394-8)
Supplement: Supplementary file 1 — Supplementary Information [file 41467_2024_49394_MOESM1_ESM.pdf]

Supplementary Information

Ligand-Mediate Exciton Allocation Enables Efficient Cluster-Based White Light-Emitting Diodes *via* Single and Heavy Doping

Jianan Sun[1], Naiyu Li[1], Zhuke Gong[1], Yi Man[1], Chunlei Zhong[1], Chunbo Duan[1], Shuo Chen[1], Jing Zhang[1], Chunmiao Han[1], Hui Xu[1,*]

[1]MOE Key Laboratory of Functional Inorganic Material Chemistry & School of Chemistry and Material Science, Heilongjiang University, 74 Xuefu Road, Harbin 150080, China.

*Correspondence to: hxu@hlju.edu.cn (HX).

Contents

Supplementary Note 1. Materials and Synthesis Method 2

Supplementary Note 2. Physical Measurements and Structures 4

Supplementary Note 3. Theoretical Simulation.. 8

Supplementary Note 4. Photophysical Analysis... 14

Supplementary Note 5. Electroluminescence Analysis.. 29

Supplementary References 53

Supplementary Note 1. Materials and Synthesis Method

Reagents and solvents for syntheses were purchased from Aldrich and Alfa-Aesar. All chemicals were used without further purification. Materials used for device fabrication were purchased from P-OLED technology company, which were used after once more sublimation.

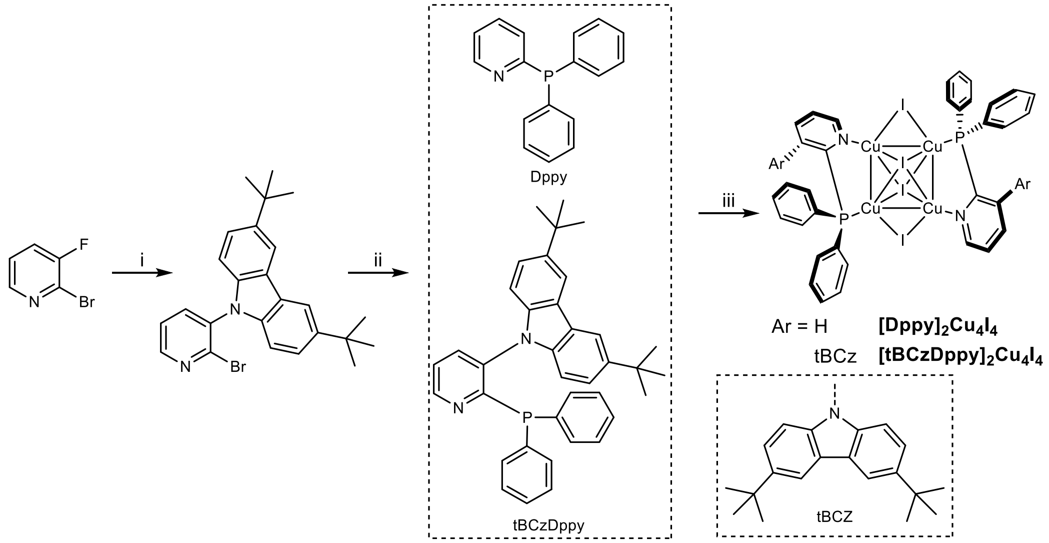

Supplementary Fig. 1 Synthetic procedures of $[Dppy]_2Cu_4I_4$ and $[tBCzDppy]_2Cu_4I_4$. i. 3,6-di-tert-butyl-carbazole, KOH, DMSO, 135 ^oC, 12 h; ii. n-BuLi, -78 ^oC, 1 h, Ph_2PCl, -78 ^oC, 1 h, room temperature, 12 h, THF; iii. CuI, DCM, room temperature, 4 h.

2-Bromo-3-*tert*-butylcarbazopyridine: 2-Bromo-3-fluoropyridine (1.00 mL, 10 mmol)) , 3,6-di-*tert*-butylcarbazole (3.07 g, 11 mmol) and potassium hydroxide (0.84 g, 15 mmol) were dissolved in dimethyl sulfoxide (DMSO, 20 mL), and the solution was stirred for 12 h at 135 ^oC under argon atmosphere. Then, the system was naturally cooled to room temperature, and the reaction was quenched by water addition. The mixture was extracted with dichloromethane, and purified with column chromatography to obtain white solid of 3.50 g with a yield of 80%. ^1H NMR (TMS, $CDCl_3$, 400 MHz): δ = 8.520 (dd, J_1 = 1.6 Hz, J_2 = 4.4 Hz, 1H), 8.152 (d, J = 1.6 Hz, 2H), 7.726 (dd, J_1 = 1.6 Hz, J_2 = 7.6 Hz, 1H), 7.457 (s, 1H), 7.432 (d, J = 2 Hz, 2H), 6.949 (d, J = 8.4 Hz, 2H), 1.457 ppm (s, 18H); ^{13}C NMR (TMS, $CDCl_3$, 100 MHz): δ = 153.1, 144.9, 140.3, 137.1, 134.8, 132.5, 115.5, 116.1, 114.1, 110.7, 34.5, 31.3 ppm; LDI-TOF: m/z (%) 434 (100) $[M^+]$; elemental analysis for $C_{25}H_{27}BrN_2$: calculated: C 68.96, H 6.25, N 6.43; found: C 68.95, H 6.24, N, 6.40.

2-Diphenylphosphine-3-*tert*-butylcarbazopyridine (tBCzDppy): In Ar, n-butyllithium (2.5 M in hexane, 4.4 mL, 11 mmol) was added into a tetrahydrofuran solution (10 mL) of 2-bromo-3-tert-butylcarbazopyridine (4.35 g, 10 mmol) at -78 ^oC in dropwise. The system was reacted under the same temperature for 1 h, and

then diphenylphosphine chloride (20 ml, 11 mmol) was added in the system in dropwise, and reacted for further 30 minutes. The mixture was then warmed to room temperature, and stirred for 12 h. After adding water, the reaction was quenched. The system was extracted with dichloromethane, and purified by column chromatography to obtain a white solid of 3.51 g with a yield of 65%. ^{1}H NMR (TMS, $CDCl_3$, 400 MHz): δ = 8.869 (d, J = 4.4 Hz, 1H), 8.066 (s, 2H), 7.624 (t, J = 7.6 Hz, 1H), 7.411 (q, J_1 = 4.4 Hz, J_2 = 7.6 Hz, 1H), 7.278 (d, J = 10.4 Hz, 2H), 7.172 (t, J = 7.2 Hz, 6H), 7.116 (t, J = 6.8 Hz, 4H), 6.715 (d, J = 8.4 Hz, 2H), 1.472 ppm (s, 18H); ^{13}C NMR (TMS, $CDCl_3$, 100 MHz): δ = 153.1, 150.4, 145.8, 140.7, 137.1, 136.2, 134.2, 133.1, 129.2, 128.7, 116.1, 115.5, 114.1, 110.7, 34.5, 31.3, ppm; LDI-TOF: m/z (%) 540 (100) [M^+]; elemental analysis for $C_{37}H_{37}N_2P$: calculated: C 82.19, H 6.90, N 5.18; found: C 82.20, H 6.92, N 5.21.

[Dppy]$_2$Cu$_4$I$_4$: In Ar, Dppy (0.26 g, 1 mmol) and CuI (0.38 g, 2 mmol) were dispersed in 5 mL of CH_2Cl_2. The mixture was stirred for 4 h at room temperature. Then, the solvent was evaporated to obtain crude material, which was further recrystallized from CH_2Cl_2/ether solution to afford yellowish crystal of 0.58 g with a yield of 90%. ^{1}H NMR (TMS, $CDCl_3$, 400 MHz): δ = 9.043 (d, J = 3.2 Hz, 2H), 7.947 (t, J = 7.6 Hz, 2H), 7.630 (t, J = 5.2 Hz, 2H), 7.438 (t, J = 5.2 Hz, 2H), 7.403 (d, J = 6.8 Hz, 4H), 7.279 ppm (q, J_1 = 6.8 Hz, J_2 = 14 Hz 16H); ^{13}C NMR (TMS, $CDCl_3$, 100 MHz): δ = 134.0, 133.8, 132.7, 132.5, 130.9, 129.5, 129.4, 129.0 ppm; ESI-MS: m/z (%) 1287.4841 (100) [M^+]; elemental analysis for $C_{34}H_{28}Cu_4I_4N_2P_2$: calculated: C 31.70, H 2.19, N 2.17; found: C 31.73, H 2.22, N 2.19.

[tBCzDppy]$_2$Cu$_4$I$_4$: In Ar, tBCzDppy (0.54 g, 1 mmol) and CuI (0.38 g, 2 mmol) were dispersed in 5 mL of CH_2Cl_2. The mixture was stirred for 4 h at room temperature. Then, the solvent was evaporated to obtain crude material, which was further recrystallized from CH_2Cl_2/ether solution to afford yellow crystal of 1.30 g with a yield of 95%. ^{1}H NMR (TMS, $CDCl_3$, 400 MHz): δ = 9.686 (s, 2H), 7.645 (s, 8H), 7.215 (d, J = 8 Hz, 4H), 6.973 (s, 6H), 6.891 (t, J = 7.2 Hz, 6H), 6.776 (d, J = 6 Hz, 4H), 6.713 (d, J = 6.4 Hz, 8H), 1.346 ppm (s, 36H); ^{13}C NMR (TMS, $CDCl_3$, 100 MHz): δ = 143.6, 140.0, 133.6, 133.5, 129.8, 127.6, 127.5, 123.7, 116.0, 109.1, 34.7, 32.1 ppm; ESI-MS: m/z (%) 1842.1839 (100.0) [M^+]; elemental analysis for $C_{74}H_{74}Cu_4I_4N_4P_2$: calculated: C 48.22, H 4.05, N 3.04; found: C 48.25, H 4.08, N 3.07. CCDC No. 2298772.

Supplementary Note 2. Physical Measurements and Structures

^1H NMR spectra were recorded using a Varian Mercury plus 400NB spectrometer relative to tetramethylsilane (TMS) as internal standard. Molecular masses were determined by a FINNIGAN LCQ Electro-Spraying Ionization-Mass Spectrometry (ESI-MS), or a MALDI-TOF-MS. Elemental analyses were performed on a Vario EL III elemental analyzer. The crystals suitable for single-crystal XRD analysis were obtained through slowly diffusing ether into dichloromethane solution of the clusters at room temperature. All diffraction data were collected at 295 K on a Rigaku Xcalibur E diffractometer with graphite monochromatized Mo Kα (λ = 0.71073 Å) radiation in ω scan mode. All structures were solved by direct method and difference Fourier syntheses. Non-hydrogen atoms were refined by full-matrix least-squares techniques on F2 with anisotropic thermal parameters. The hydrogen atoms attached to carbons were placed in calculated positions with C−H = 0.93 Å and U(H) = 1.2Ueq(C) in the riding model approximation. All calculations were carried out with the SHELXL97 program. Thermogravimetric analysis (TGA) and differential scanning calorimetry (DSC) were performed on Shimadzu DSC-60A and DTG-60A thermal analyzers under nitrogen atmosphere at a heating rate of 10 °C min^{-1}. Cyclic voltammetric (CV) studies were conducted using an Eco Chemie B. V. AUTOLAB potentiostat in a typical three-electrode cell with a glassy carbon working electrode, a platinum wire counter electrode, and a silver/silver chloride (Ag/AgCl) reference electrode. Absorption spectra were measured using a SHIMADZU UV-3150 spectrophotometer.

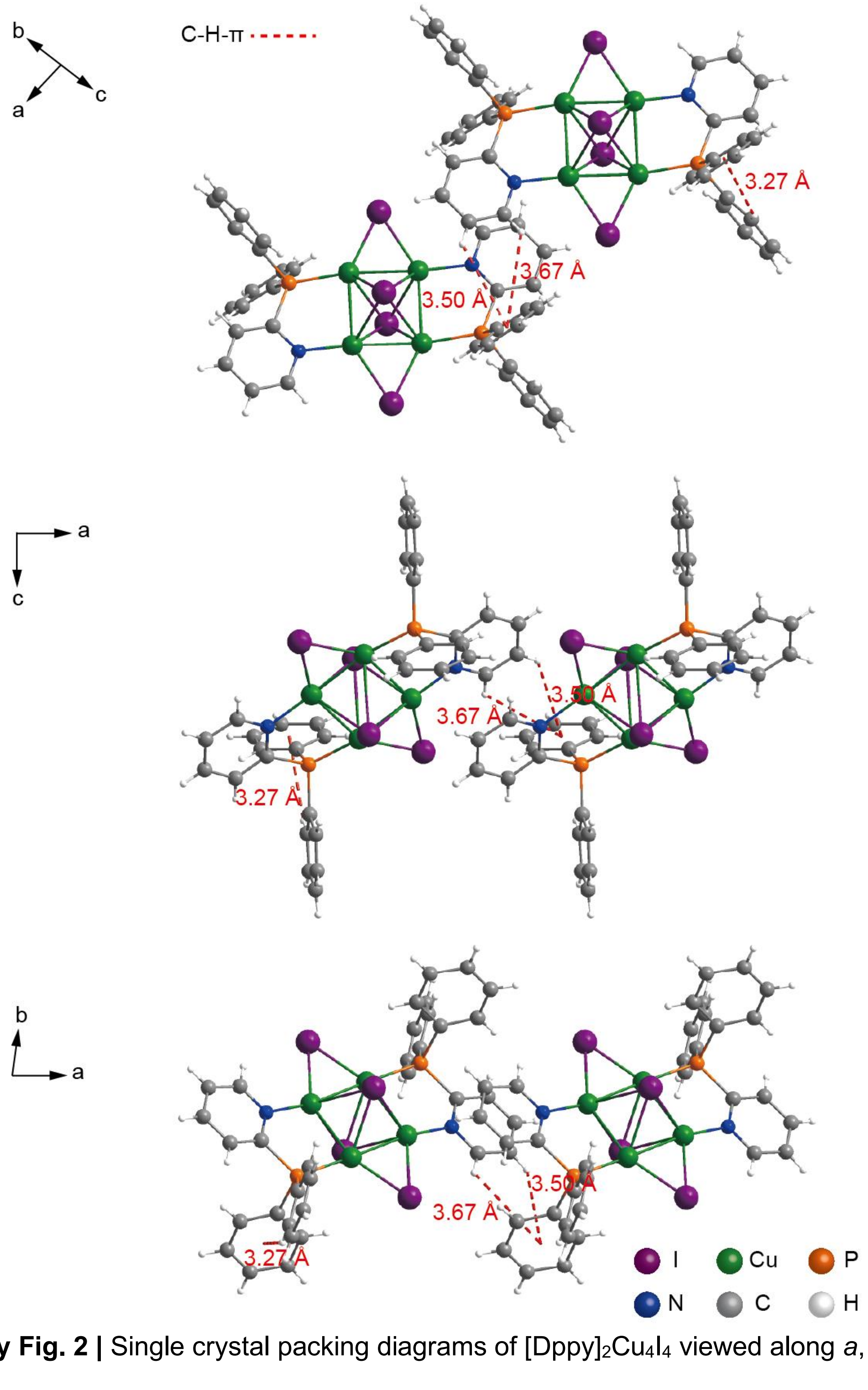

Supplementary Fig. 2 | Single crystal packing diagrams of $[Dppy]_2Cu_4I_4$ viewed along *a*, *b* and *c* axes.

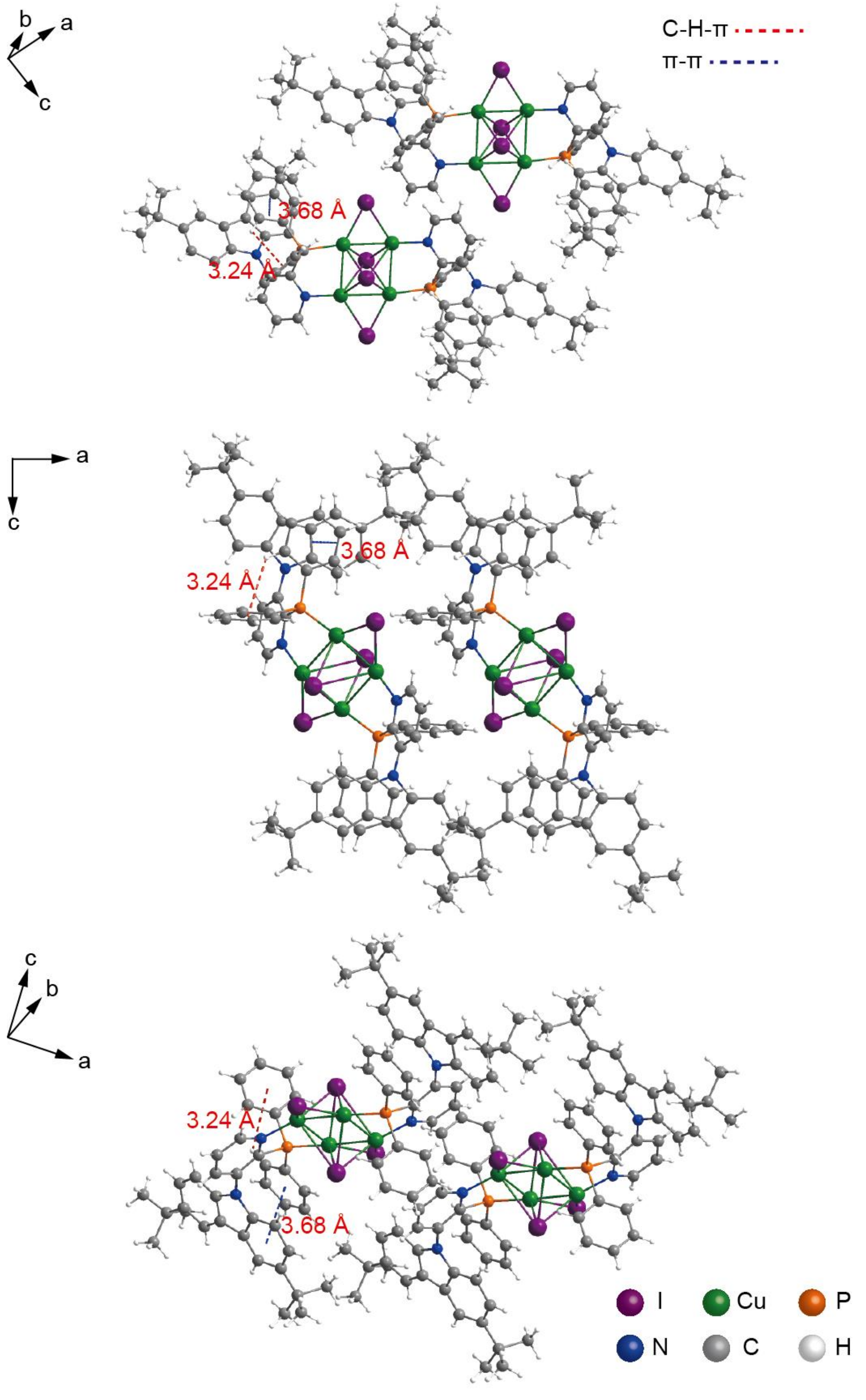

Supplementary Fig. 3 | Single crystal packing diagrams of $[tBCzDppy]_2Cu_4I_4$ viewed along *a*, *b* and *c* axes.

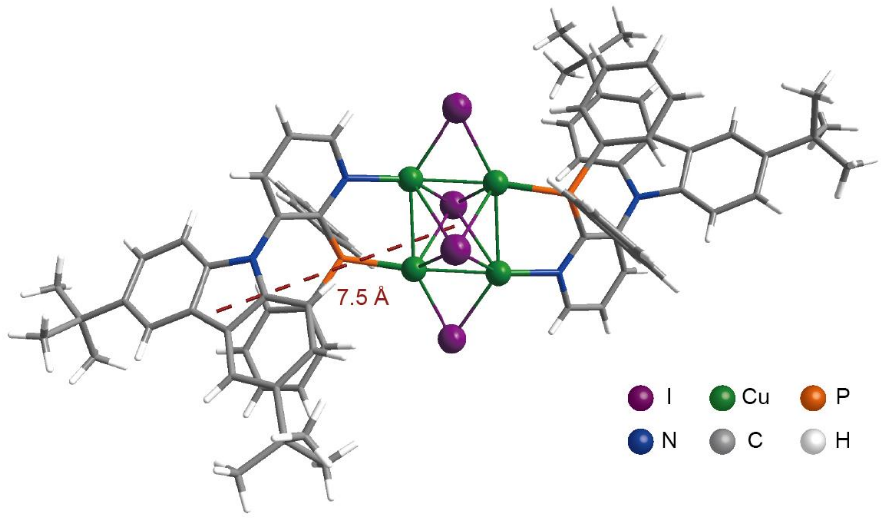

Supplementary Fig. 4 | Single crystal structure of [tBCzDppy]$_2$Cu$_4$I$_4$. The brown dash line indicates the centroid-centroid distances of tBCz and Cu$_4$I$_4$ core.

Supplementary Note 3. Theoretical Simulation

Density functional theory (DFT) and time-dependent DFT (TDDFT) computations were carried out with different parameters for structure optimizations and vibration analyses. The ground state (S_0) configuration was established according to single crystal data. The S_0, singlet and triplet states in vacuum were simulated by the restricted and unrestricted formalism of Beck's three-parameter hybrid exchange functional[1] and Lee, and Yang and Parr correlation functional[2] B3LYP/6-31G(d,p) for ligands and double-ζ LANL2DZ basis sets for CuI, respectively. The fully optimized stationary points were further characterized by harmonic vibrational frequency analysis to ensure that real local minima had been found without imaginary vibrational frequency. The total energies were also corrected by zero-point energy both for the ground state and triplet state. Natural transition orbital (NTO) analysis was performed on the basis of optimized ground-state geometries at the same level.[3] The contours were visualized with Gaussview 5.0. All computations were performed using the Gaussian 09 package.[4]

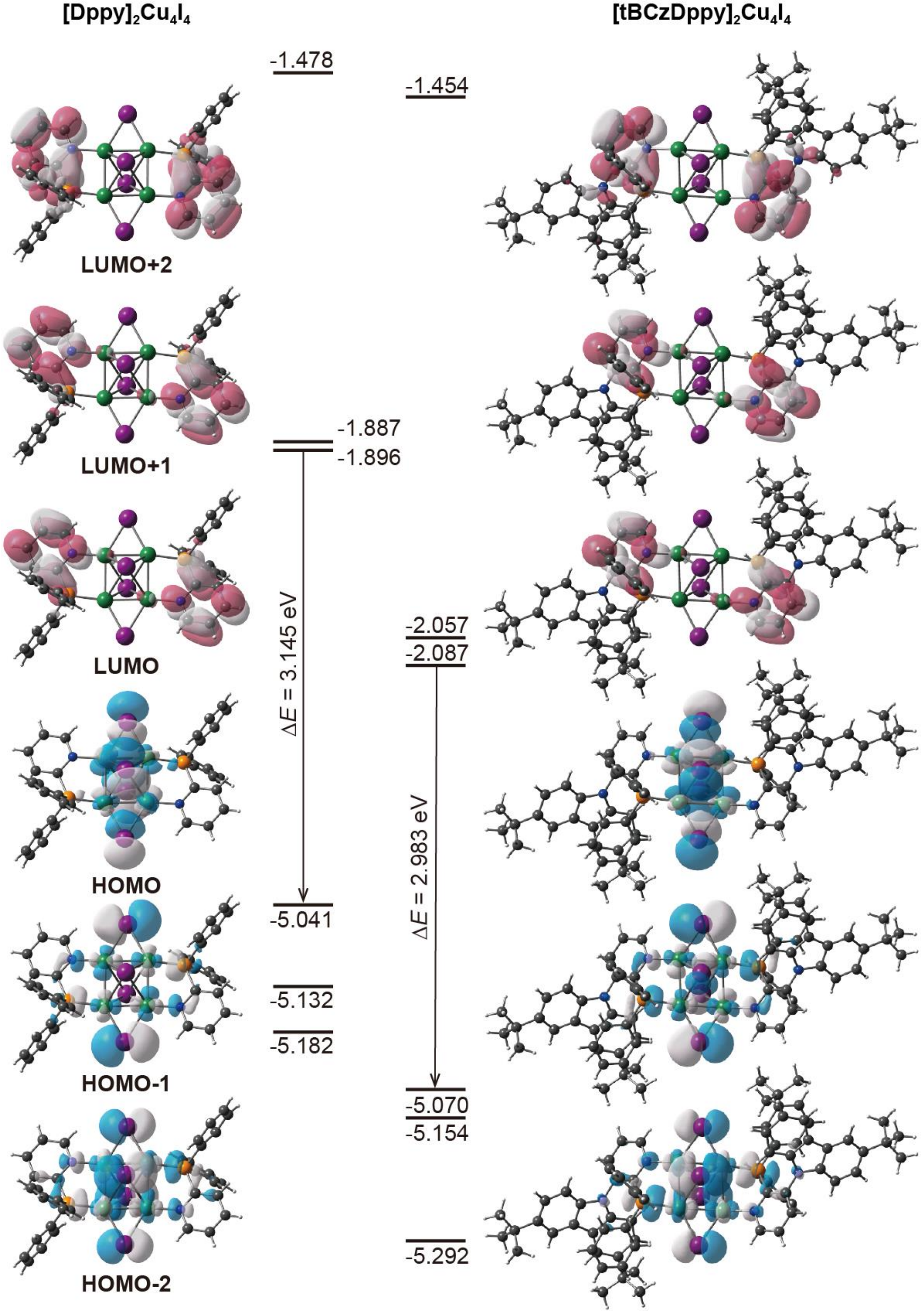

Supplementary Fig. 5 | FMO energy levels and contours of $[Dppy]_2Cu_4I_4$ and $[tBCzDppy]_2Cu_4I_4$ simulated at the level of B3LYP/6-31G*. The seperated occupied and unoccupied molecular oribtals indicate the predominance of charge transfer interactions between ligands and metallic cores. Energy gap between HOMO and HOMO-4 of $[tBCzDppy]_2Cu_4I_4$ is within 0.4 eV.

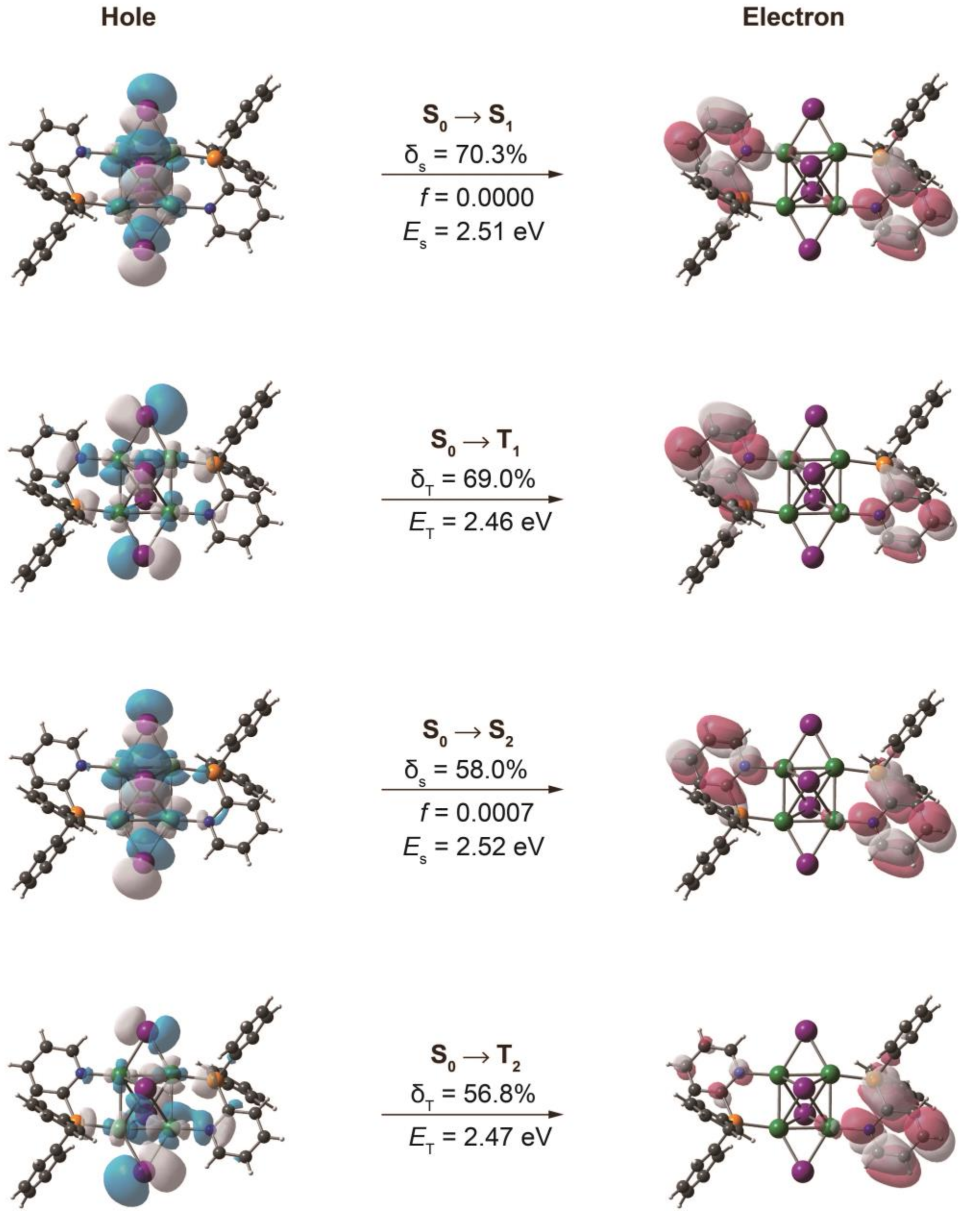

Supplementary Fig. 6 | Contours and key transition parameters of the $S_0 \rightarrow S_n$ and $S_0 \rightarrow T_n$ (n = 1 and 2) excitations for $[Dppy]_2Cu_4I_4$ simulated with natural transition orbital (NTO) analysis at the level of B3LYP/6-31G*.

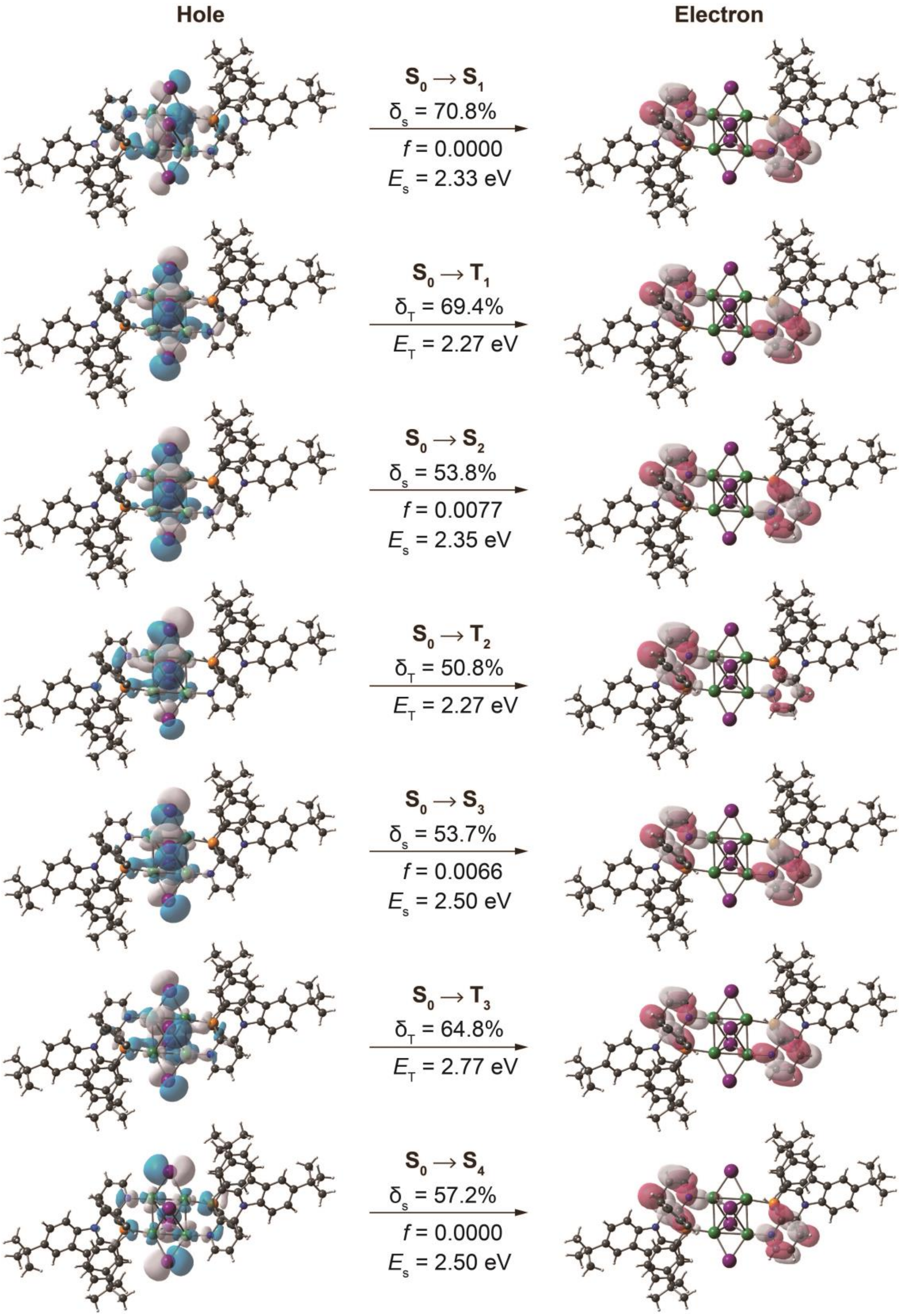

Supplementary Fig. 7 | Contours and key transition parameters of the $S_0 \rightarrow S_n$ and $S_0 \rightarrow T_n$ (n = 1-5, 9 and 10) excitations for $[tBCzDppy]_2Cu_4I_4$ simulated with NTO analysis at the level of B3LYP/6-31G*.

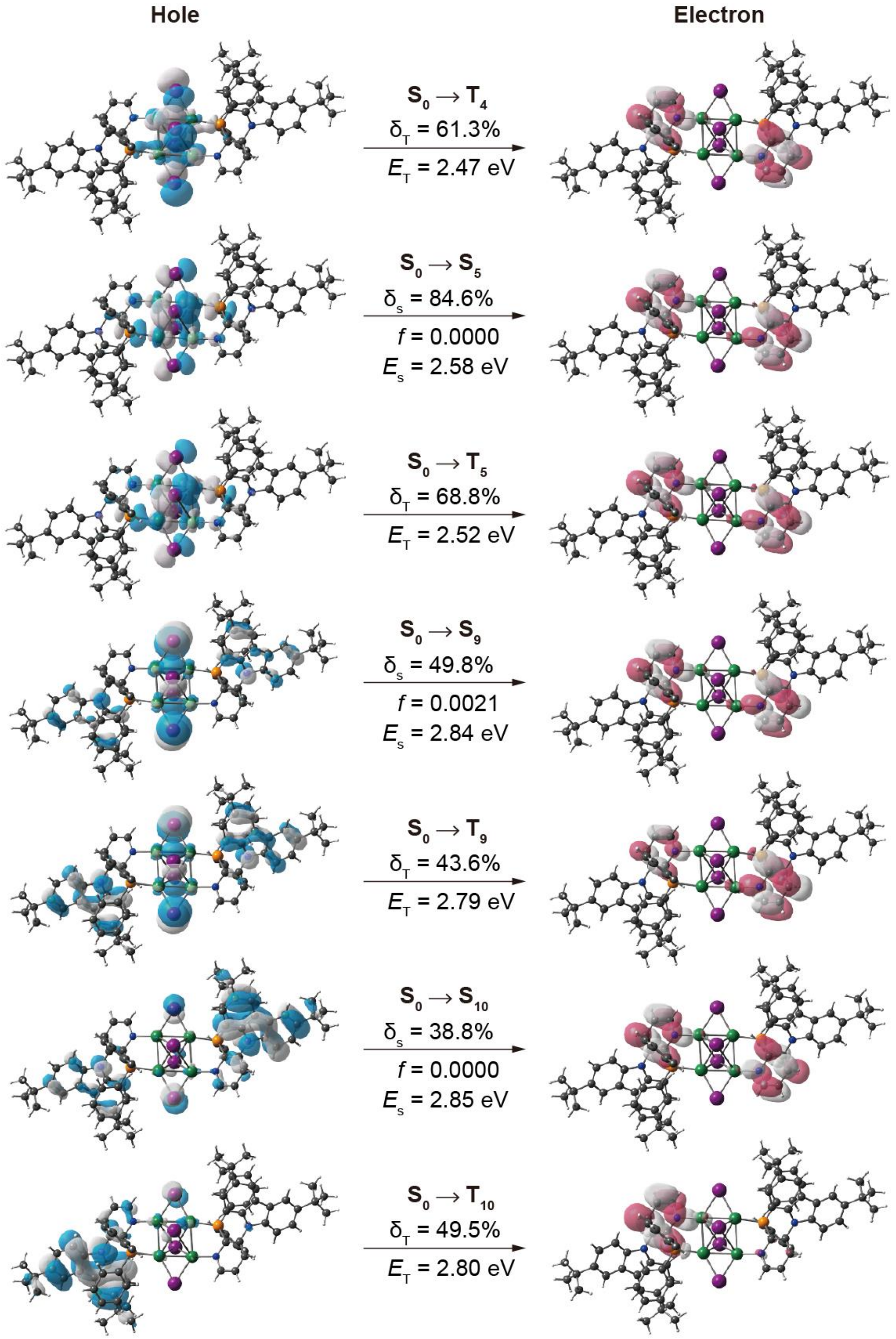

Supplementary Fig. 8 | Contours and key transition parameters of the $S_0 \rightarrow S_n$ and $S_0 \rightarrow T_n$ (n = 1-5, 9 and 10) excitations for $[tBCzDppy]_2Cu_4I_4$ simulated with NTO analysis at the level of B3LYP/6-31G*.

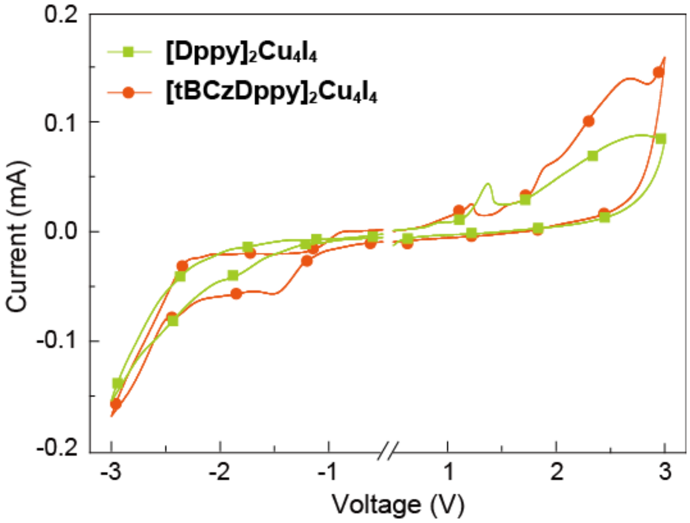

Supplementary Fig. 9 | CV curves of $[Dppy]_2Cu_4I_4$ and $[tBCzDppy]_2Cu_4I_4$ measured at room temperature with the scanning rate of 100 mV s^{-1}.

Supplementary Note 4. Photophysical Analysis

1. Spectral measurement

Steady-state emission spectra were measured using an Edinburgh FPLS 1000 fluorescence spectrophotometer. Films were prepared by spin coating. Time-resolved emission spectra were measured with Time-Correlated Single Photon Counting (TCSPC) method with a nanosecond hyrogen flash lamp and a microsecond pulsed Xenon light source for 100 ps-10 s lifetime measurement, the synchronization photomultiplier for signal collection and the Multi-Channel Scaling Mode of the PCS900 fast counter PC plug-in card for data processing. A temperature controller was equipped to achieve 11-500 K variation. Spectra of prompt fluorescence (PF), delayed fluorescence (DF) and phosphorescence (PH) were sliced from time-resolved emission spectra (TRES) in the time ranges of <1 μs, 1-100 μs and >150 μs, respectively.

2. Photoluminescence quantum yield measurement

Photoluminescence quantum yields (PLQY, ϕ_{PL}) of these films were measured through a Labsphere 1-M-2 (ϕ = 6") integrating sphere coated with Benflect having efficient light reflection from 200-1600 nm, which was integrated with FPLS 1000. The absolute ϕ_{PL} determination of the sample was performed with two spectral (emission) scans, with the emission monochromator scanning over the Rayleigh scattered light from the sample and a blank substrate. The first spectrum recorded the scattered light and the sample emission, and the second spectrum recorded the scattered light of the Benflect coating. Integration and subtraction of the scattered light in the two spectra are equal to the number of photons absorbed by the samples (N_a), while integration of the sample emission is equal to the number of photons emitted (N_e). Then, absolute ϕ_{PL} can be estimated according to the equation of ϕ_{PL} = N_e/N_a. Spectral correction (emission arm) was applied to raw data after background subtraction, and from these spectrally corrected curves, the quantum yield was calculated using an F900 software wizard.

3. Transition Parameter Calculation

The calculation formulas for the rate constants of prompt fluorescence (k_{PF}), delayed fluorescence (k_{DF}), singlet radiation (k_r^S),singlet (k_{nr}^S) and triplet nonradiation (k_{nr}^T), reverse intersystem crossing (k_{RISC}) and intersystem crossing (k_{ISC}), and corresponding quantum efficiencies (ϕ) are expressed as following list:[6-7]

$$k_{\mathrm{PF}} = k_r^S + k_{nr}^S + k_{\mathrm{ISC}} \quad \text{(Eq. S1)}$$

$$k_{\mathrm{DF}} = k_{nr}^T + \left(1 - \frac{k_{\mathrm{ISC}}}{k_{\mathrm{PF}}}\right) \cdot k_{\mathrm{RISC}} \quad \text{(Eq. S2)}$$

$$\mathrm{k_r^S = \phi_{PF} \cdot k_{PF}} \quad \text{(Eq. S3)}$$

$$k_{nr}^S = k_{\mathrm{PF}} - k_r^S - k_{\mathrm{ISC}} = k_{\mathrm{PF}} - k_r^S - k_{\mathrm{PF}} \cdot \frac{\eta_{\mathrm{PF}}}{\eta_{\mathrm{PL}}} \quad \text{(Eq. S4)}$$

$$k_{\mathrm{ISC}} = (1 - \phi_{PF}) \cdot k_{\mathrm{PF}} \quad \text{(Eq. S5)}$$

$$\mathrm{k_{nr}^{T}} = \mathrm{k_{DF}}\text{-}\left(1\text{-}\frac{\mathrm{k_{ISC}}}{\mathrm{k_{PF}}}\right) \cdot \mathrm{k_{RISC}} = \mathrm{k_{DF}}\text{-}\left(1\text{-}\frac{\mathrm{k_{ISC}}}{\mathrm{k_{PF}}}\right) \cdot \frac{\mathrm{k_{DF}} \cdot \mathrm{k_{PF}} \cdot \phi_{\mathrm{DF}}}{\mathrm{k_{ISC}} \cdot \phi_{\mathrm{PF}}} \quad \text{(Eq. S6)}$$

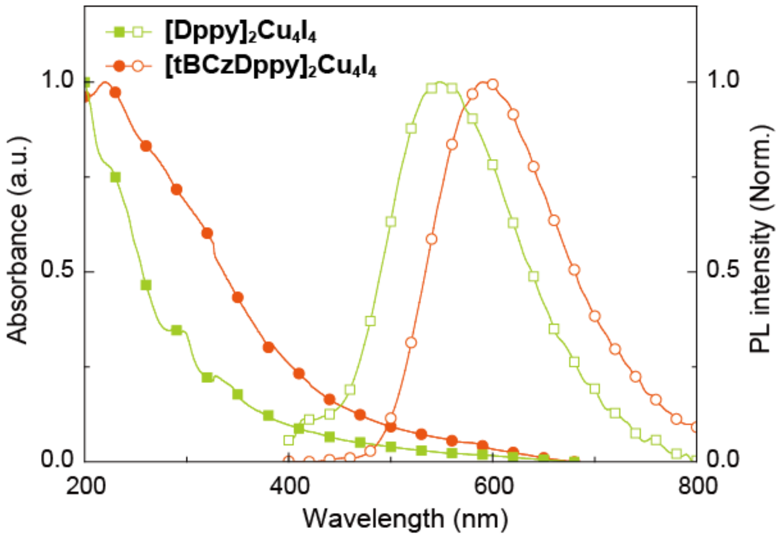

Supplementary Fig. 10 | Electronic absorption spectra and emission spectra of spin-coated neat films for $[Dppy]_2Cu_4I_4$ and $[tBCzDppy]_2Cu_4I_4$.

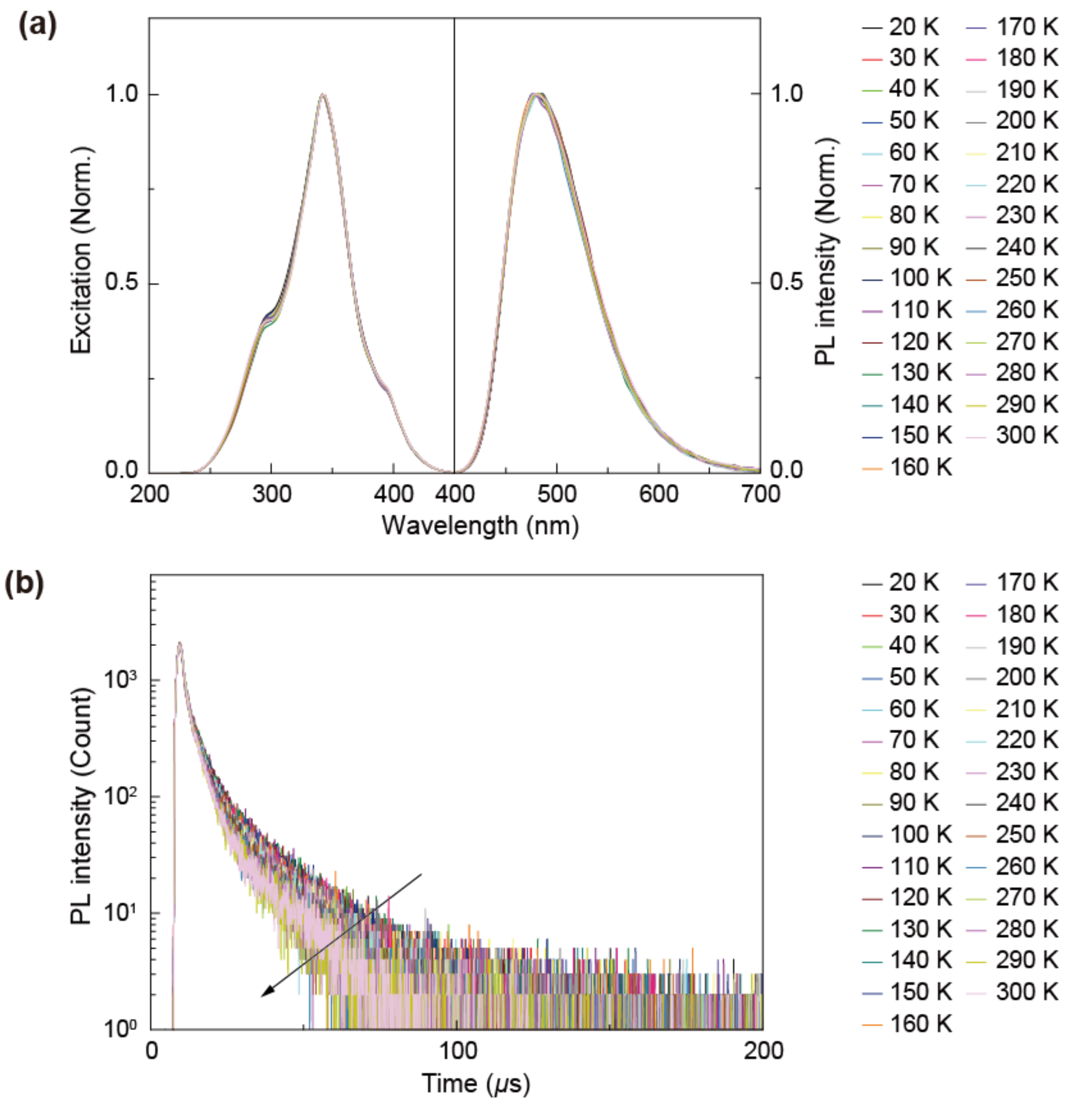

Supplementary Fig. 11 | Excitation and PL spectra and time decays of neat CzAcSF film at different temperatures. (a) Excitation spectra (left) and emission spectra (right). (b) Time decays.

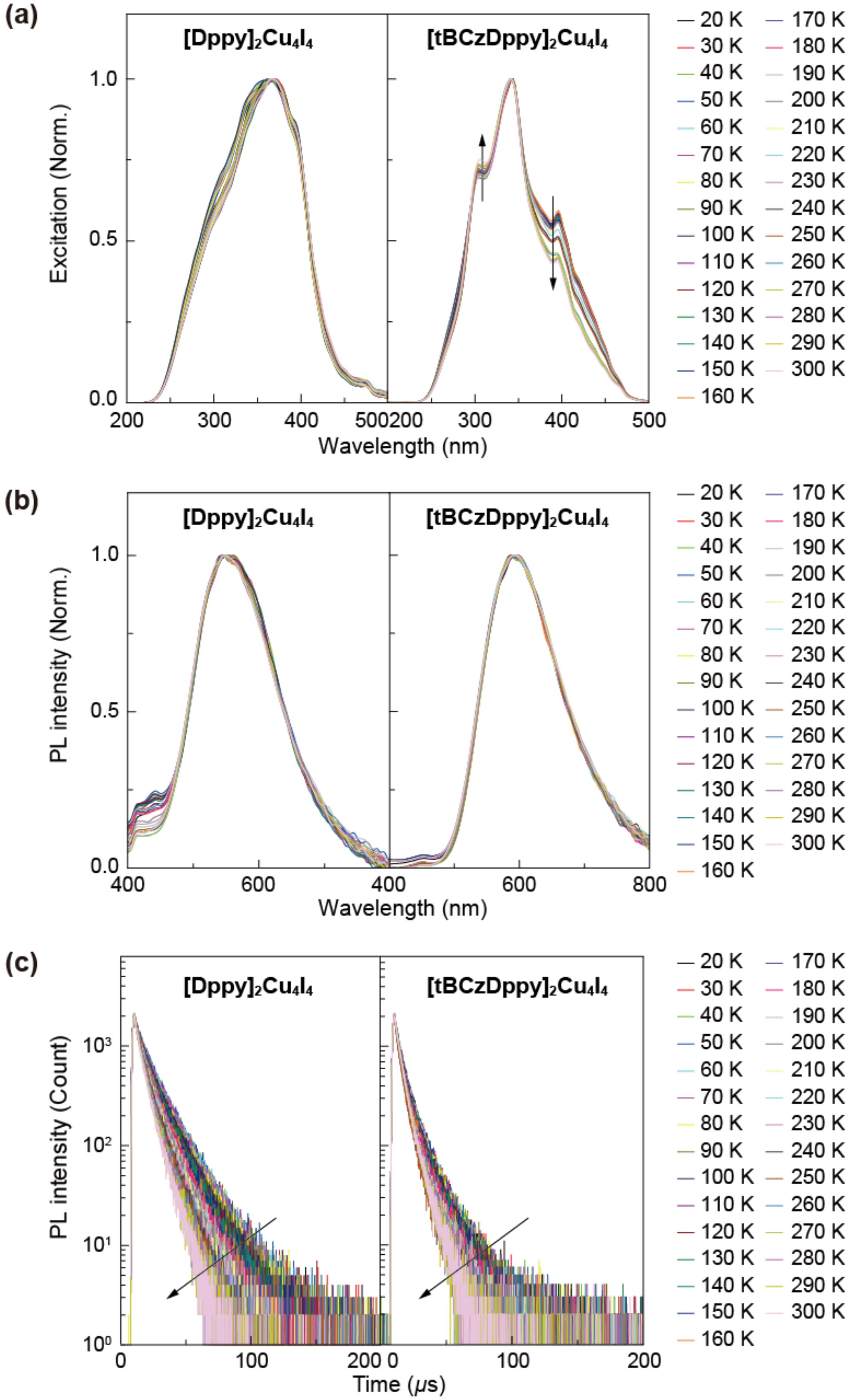

Supplementary Fig. 12 | Excitation and PL spectra and time decays of neat **[Dppy]$_2$Cu$_4$I$_4$** and **[tBCzDppy]$_2$Cu$_4$I$_4$** films at different temperatures. (a) Excitation spectra. (b) Emission spectra. (c) Time decays.

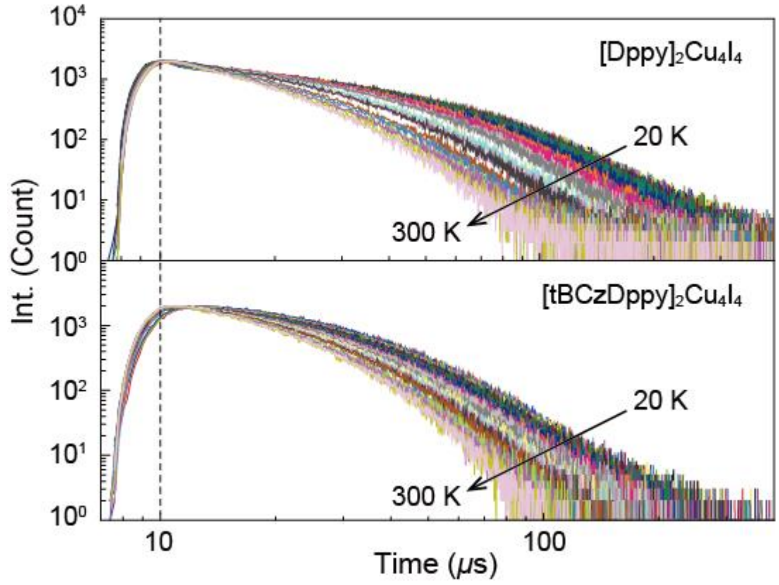

Supplementary Fig. 13 | Time decays of **$[Dppy]_2Cu_4I_4$** and **$[tBCzDppy]_2Cu_4I_4$** powders in the range of 20-300 K with an interval of 10 K.

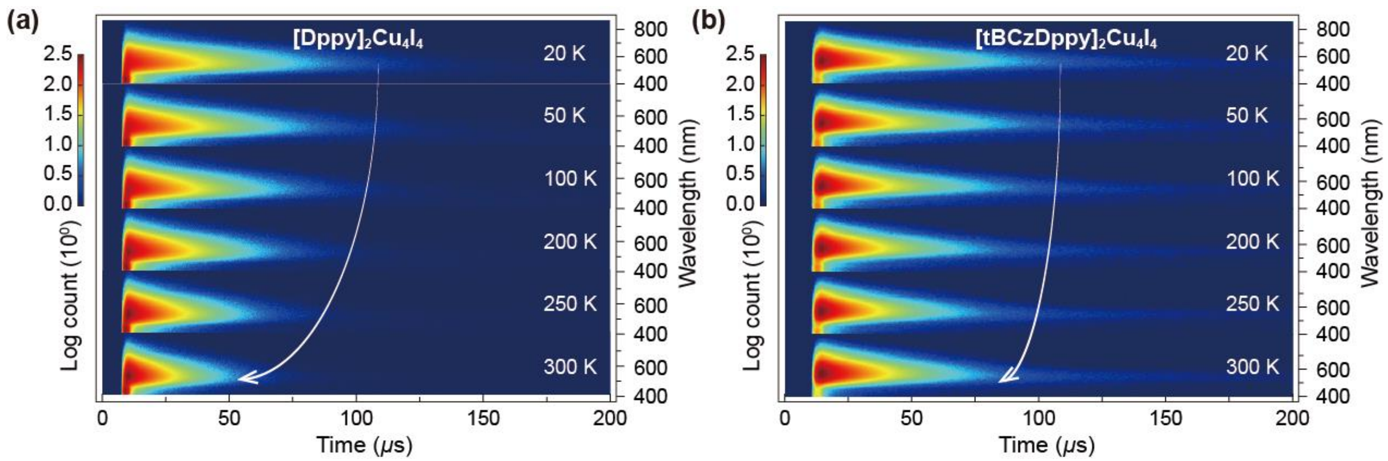

Supplementary Fig. 14 | Time-resolved emission spectra of neat films at different temperatures. (a) $[Dppy]_2Cu_4I_4$. (b) $[tBCzDppy]_2Cu_4I_4$.

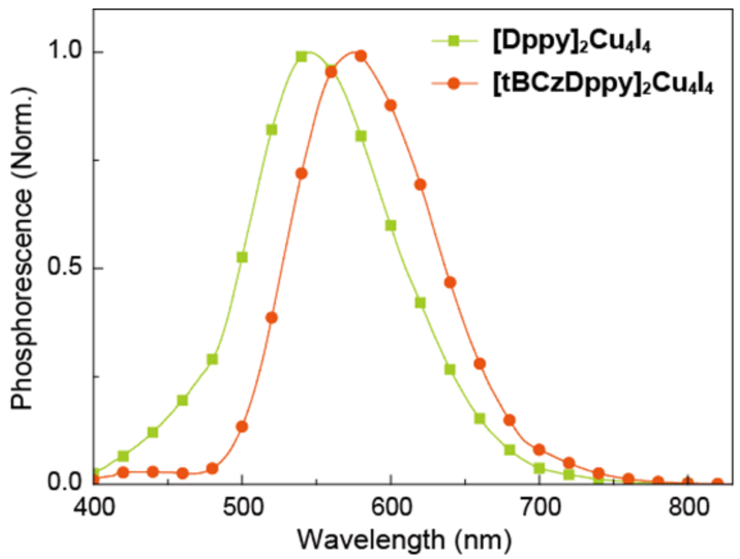

Supplementary Fig. 15 | Time-resolved phosphorescence spectra after 100 μs of (a) **[Dppy]$_2$Cu$_4$I$_4$** and (b) **[tBCzDppy]$_2$Cu$_4$I$_4$** based spin-coating neat films.

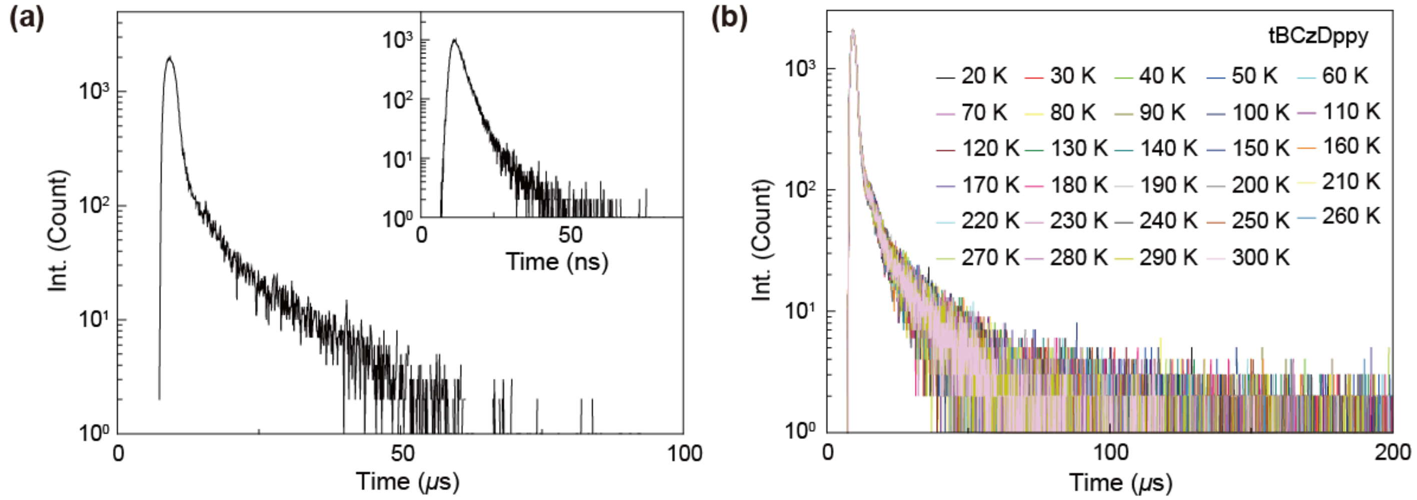

Supplementary Fig. 16 | Time decays of spin-coated film based on neat tBCzDppy. (a) At microsecond and nanosecond (inset) scale at room temperature. (b) In the range of 20-300 K with an interval of 10 K.

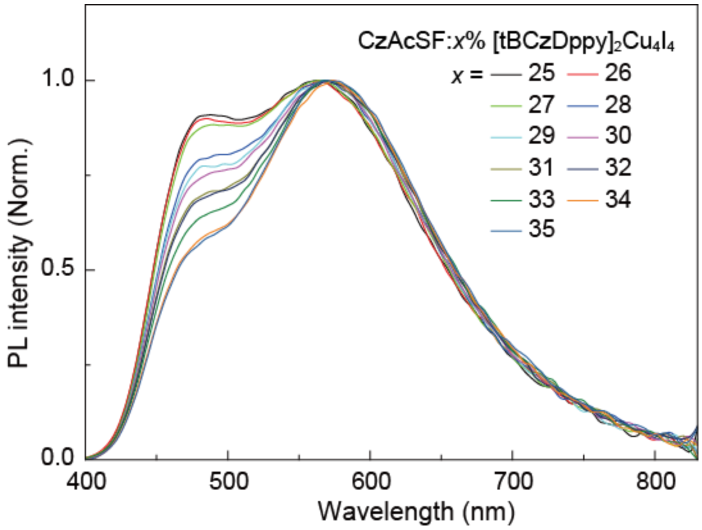

Supplementary Fig. 17 | PL spectra of CzAcSF:*x*% $[tBCzDppy]_2Cu_4I_4$ films in the range of *x* = 25-35 with an interval of 1%.

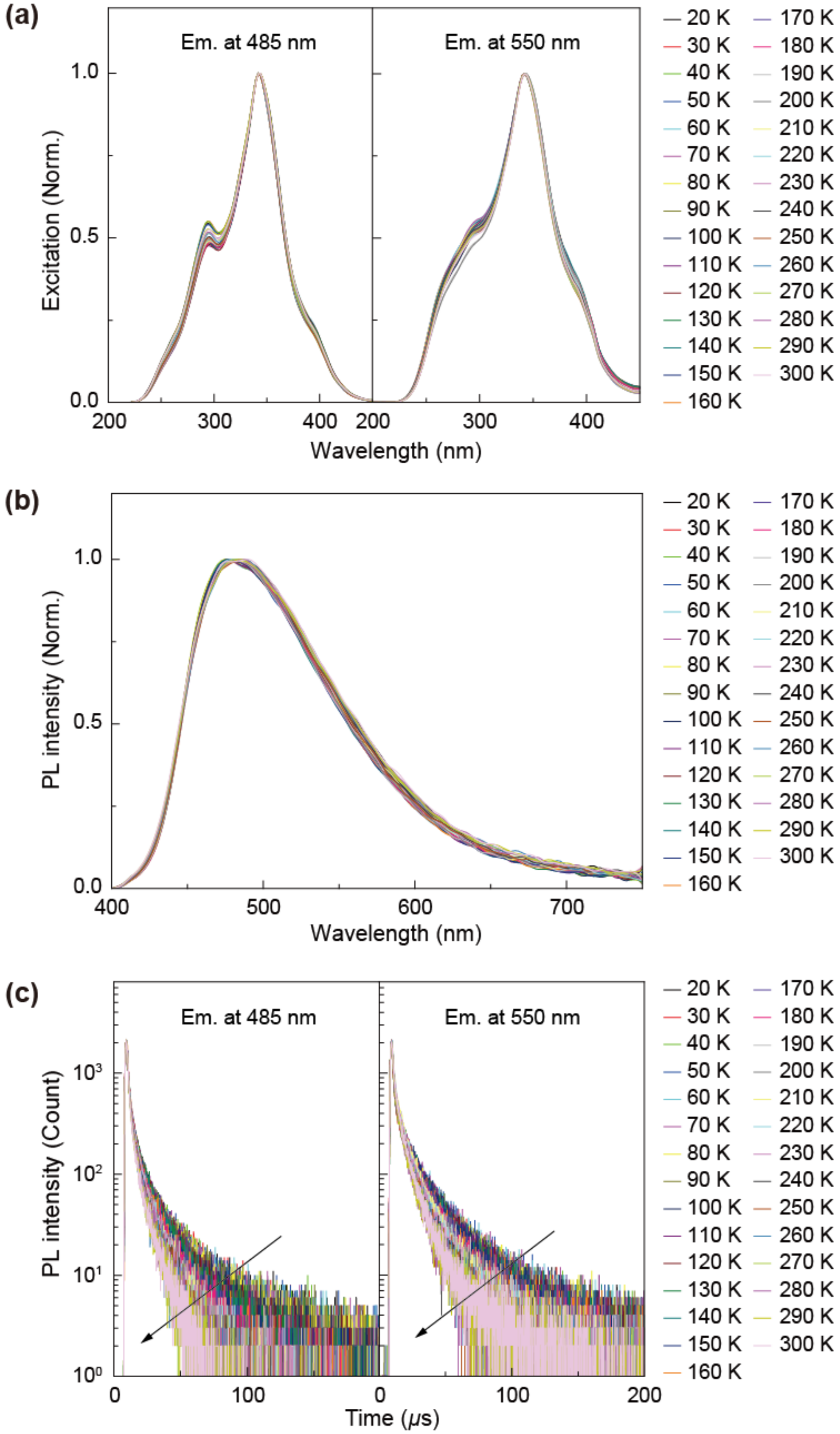

Supplementary Fig. 18 | Excitation and PL spectra and time decays of spin-coated CzAcSF:30% $[Dppy]_2Cu_4I_4$ film in the range of 20-300 K with an interval of 10 K. (a) Excitation spectra at emission wavelengths at 485 nm (left) and 550 nm (right). (b) Emission spectra. (c) Time decays at emission wavelength at 485 nm (left) and 550 nm (right).

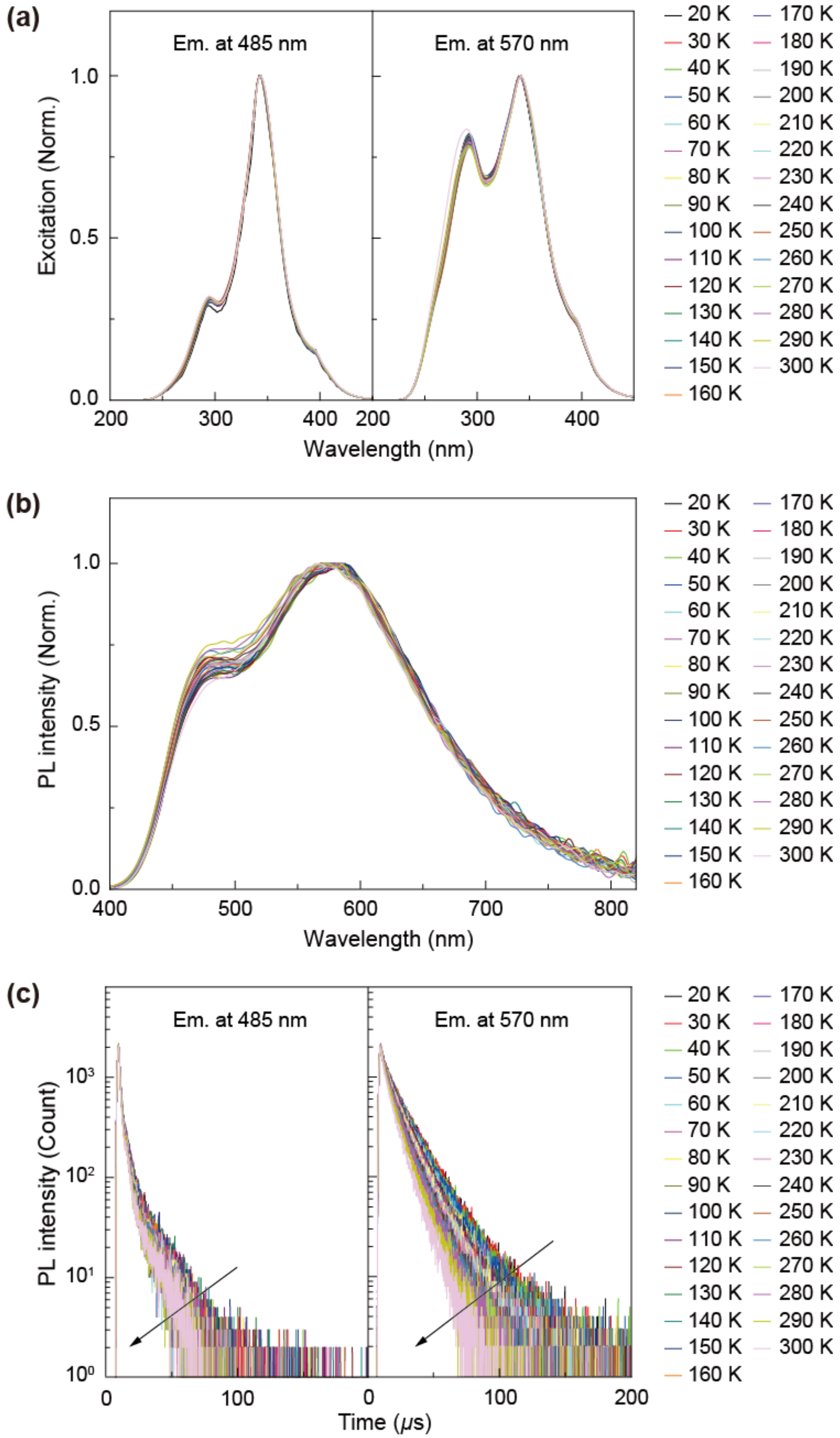

Supplementary Fig. 19 | Excitation and PL spectra and time decays of spin-coated CzAcSF:30% $[tBCzDppy]_2Cu_4I_4$ film in the range of 20-300 K with an interval of 10 K. (a) Excitation spectra at emission wavelengths at 485 nm (left) and 570 nm (right). (b) Emission spectra. (c) Time decays at emission wavelength at 485 nm (left) and 570 nm.

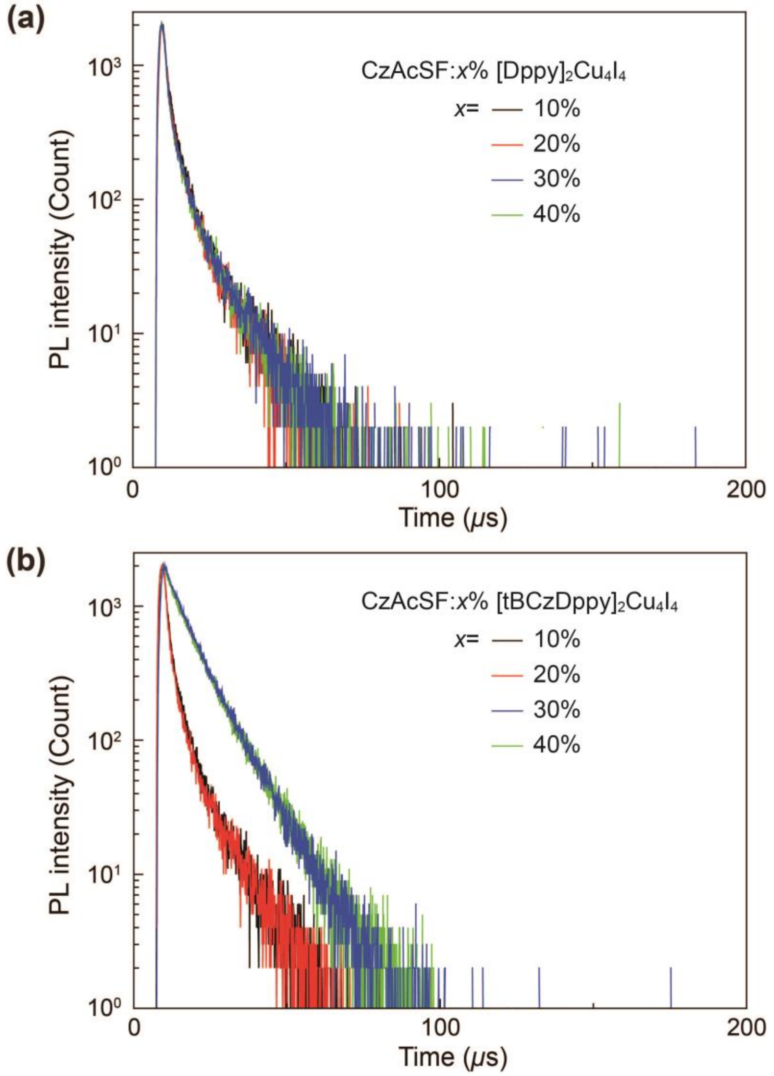

Supplementary Fig. 20 | Time decays of CzAcSF: *x*% Cu_4I_4 cluster films (*x* = 10, 20, 30 and 40) at 550 and 570 nm, repectively, at room temperature. (a) $[DPPy]_2Cu_4I_4$. (b) $[tBCzDppy]_2Cu_4I_4$.

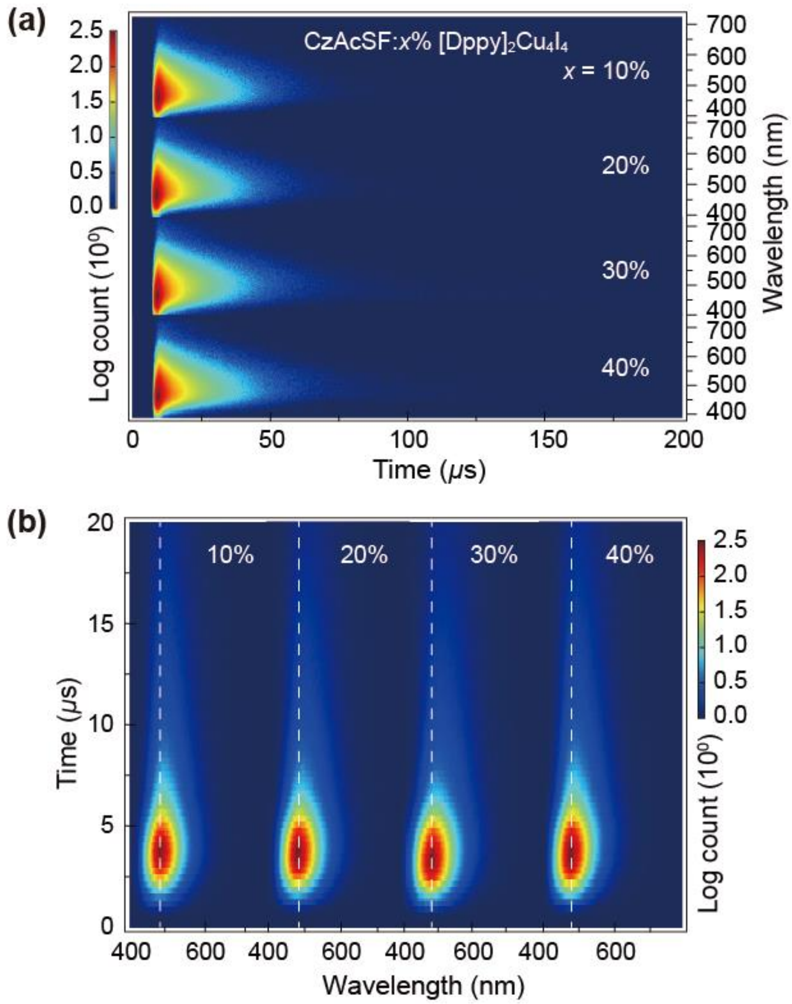

Supplementary Fig. 21 | Photophysical properties of CzAcSF: *x*% $[Dppy]_2Cu_4I_4$ (*x* = 10, 20, 30 and 40) films.(a) Time-resolved emission spectra. (b) Sliced TRES contours during the first 40 *μ*s.

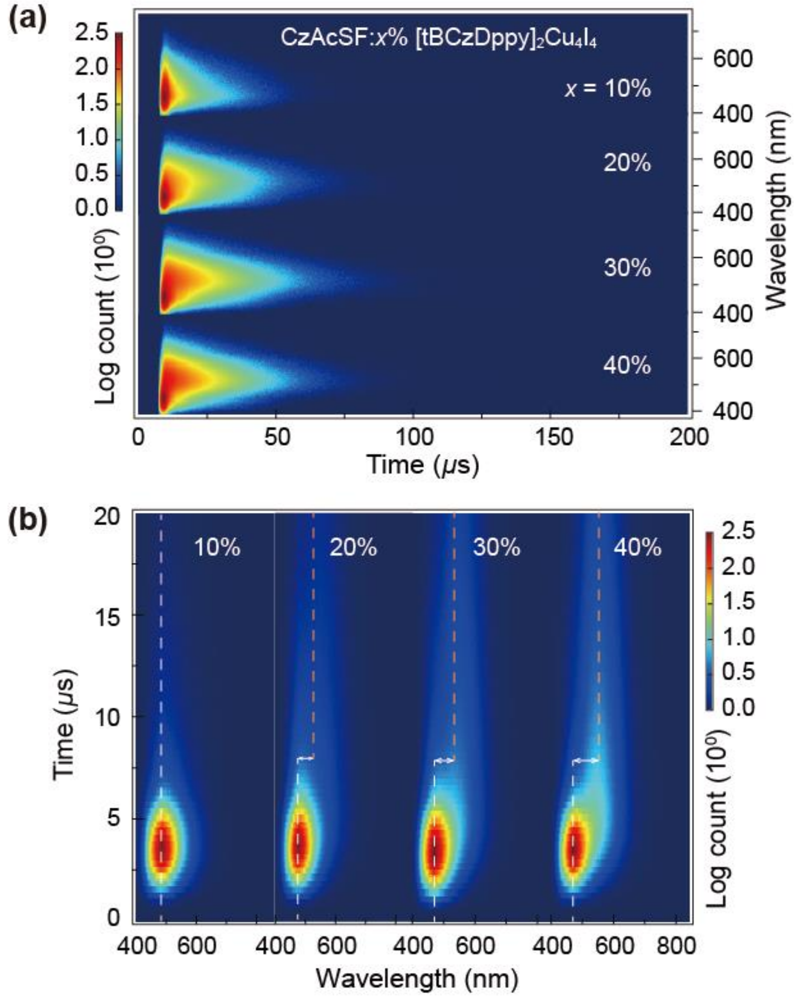

Supplementary Fig. 22 | Photophysical properties of CzAcSF: *x*% $[tBCzDppy]_2Cu_4I_4$ (*x* = 10, 20, 30 and 40) films.(a) Time-resolved emission spectra. (b) Sliced TRES contours during the first 40 *μ*s.

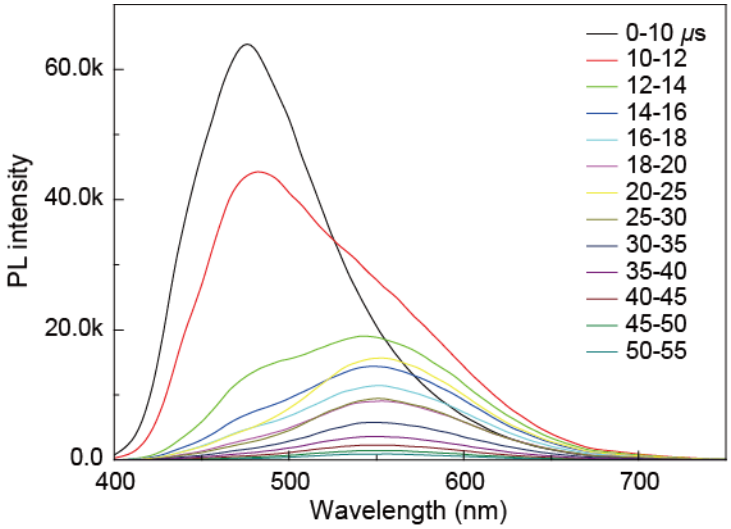

Supplementary Fig. 23 | Sliced time resolved emission spectra (TRES) of CzAcSF:30% $[tBCzDppy]_2Cu_4I_4$ film at room temperature in the time range of 0-55 μs.

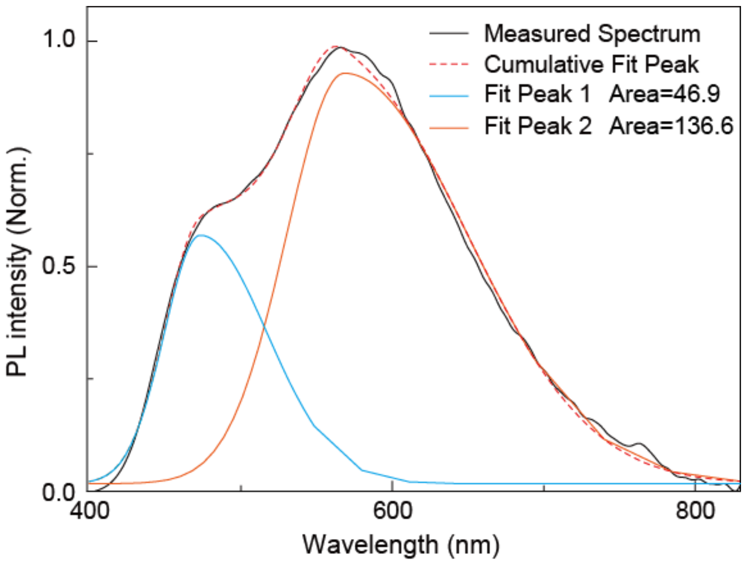

Supplementary Fig. 24 | Double-peak fitting of PL profile for CzAcSF:30% $[tBCzDppy]_2Cu_4I_4$ film and corresponding blue and yellow peak areas for PLQY estimation.

Supplementary Table 1. Physical properties of the clusters.

Cluster	λ_{Abs}[a] (nm)	λ_{PL}[b] (nm)	S_1 (eV)	T_1 (eV)	ΔE_{ST}[f] (eV)	τ[g] (μs)	PLQY (%)	HOMO (eV)	LUMO (eV)	FWHM[j] (nm)
**$[Dppy]_2Cu_4I_4$**	275, 347	548	2.33[c], 2.51[d]	2.28[e], 2.46[d]	0.05	2.95 (0.68), 8.80 (0.32)	1[b], 10[h]	-5.04[d], -5.98[i]	-1.90[d], -3.25[i]	148
**$[tBCzDppy]_2Cu_4I_4$**	297,328, 342	590	2.34[c], 2.33[d]	2.15[e], 2.27[d]	0.19	6.66 (0.31), 6.85 (0.69)	37[b], 80[h]	-5.07[d], -5.71[i]	-2.09[d], -3.16[i]	147

[a] In DCM solution (10^{-6} mol L^{-1}); [b] in neat film; [c] estimated according to absorption edge; [d] Gaussian simulation results of single molecules; [e] estimated according to peak wavelengths of time-resolved phosphorescence spectra (Supplementary Fig. 15) after a delay of 100 μs; [f] singlet-triplet splitting; [g] lifetimes fitted according to time decays. Data in parentheses are percentages; [h] in polymethyl methacrylate (PMMA) film; [i] calculated according to onset voltages of cyclic voltammetric curves; [j] full width at half maximum.

Supplementary Table 2. Key transition parameters of CzAcSF:*x*% cluster films.

Cluster	*x*	ϕ_{PL}[a] (%)	λ_{Em} (nm)	ϕ_{PL} (%)	τ_{PF}[d] (ns)	τ_{DF}[e] (μs)	ϕ_{PF}[f] (%)	ϕ_{DF}[g] (%)	k_{PF}[h] (10^7 s^{-1})	k_{DF}[i] (10^4 s^{-1})	k_{ISC}[j] (10^6 s^{-1})	k_{RISC}[k] (10^4 s^{-1})	k_r^S [l] (10^6 s^{-1})	ϕ_{ISC}[m] (%)	ϕ_{RISC}[n] (%)
$[Dppy]_2Cu_4I_4$	10	29	484	29[b]	16.4	4.4	14	15	0.88	3.39	4.51	6.82	1.26	51	74
	20	28	488	28[b]	14.7	4.6	14	15	0.92	3.26	4.92	6.76	1.24	54	74
	30	22	490	22[b]	18.3	4.8	10	11	0.57	2.35	2.92	4.98	0.59	51	73
	40	19	490	19[b]	13.0	5.1	10	9	0.76	1.76	3.61	3.39	0.75	47	70
$[tBCzDppy]_2Cu_4I_4$	10	79	478	36[b]	8.6	2.2	26	10	3.03	4.64	8.60	6.39	7.92	28	68
			544	43[c]	19.9	4.2	23	20	1.00	4.79	5.33	9.07	2.59	47	77
	20	81	482	30[b]	12.7	3.3	20	11	2.00	3.18	5.48	4.80	3.12	35	68
			556	51[c]	16.6	4.0	26	25	1.56	6.18	7.56	12.16	4.04	48	80
	30	82	488	21[b]	13.8	3.4	12	8	0.88	2.44	3.49	4.20	1.08	40	69
			569	61[c]	16.5	8.8	16	46	0.95	5.20	7.14	20.22	1.49	75	91
	40	76	480	15[b]	5.2	2.5	11	4	2.10	1.76	6.15	2.42	2.28	29	62
			576	61[c]	13.8	8.9	16	45	1.15	5.08	8.54	19.48	1.83	74	91

[a] Absolute PL quantum yield (PLQY) of whole white emission measured with integrating sphere; PLQY of [b] blue and [c] yellow components, which were estimated according to blue and yellow ratios in whole white emission profiles (Supplementary Fig. 24); lifetimes of [d] prompt fluorescence (PF) and [e] delayed fluorescence (DF); efficiencies of [f] PF and [g] DF; rate constants of [h] PF, [i] DF, [j] intersystem crossing (ISC), [k] reverse ISC (RISC) and [l] singlet radiation; efficiencies of [m] ISC [m] and [n] RISC.

Supplementary Note 5. Electroluminescence Analysis

1. Device fabrication

Before loading into a deposition chamber, the ITO substrate was cleaned with detergent and deionized water, dried in an oven at 120 °C for 4 h, and treated with oxygen plasma for 3 min. Poly(3,4-ethylenedioxythiophene): poly(styrenesulfonate) (PEDOT:PSS) layer (~40 nm) as hole transporting layer was spin-coated on the substrate. Then, cluster-doped emissive layers were spin-coated on PEDOT:PSS layer. CzAcSF, BCPO and clusters were dissolved in chlorobenzene with the total concentrations of 10 mg ml^{-1}. The substrate was transferred to ultrahigh-vacuum chamber, and then electron transporting layers were evapored at a rate of 0.1-0.2 nm s^{-1} sequentially at a pressure below 4×10^{-4} Pa. Onto the electron-transporting layer, a layer of LiF with 1-nm thickness was deposited at a rate of 0.1 nm s^{-1} to improve electron injection. Finally, a 100-nm layer of Al was deposited at a rate of 0.6 nm s^{-1} as the cathode. The emission area of the devices was 0.09 cm^2, as determined by the overlapped area of the anode and the cathode. After fabrication, devices were immediately transferred to a glove box for encapsulation with glass coverslips using epoxy glue. The devices were firstly applied with bias from zero to find out the turn-on voltages, and then formally measured from the turn-on voltages to minimize operation errors.

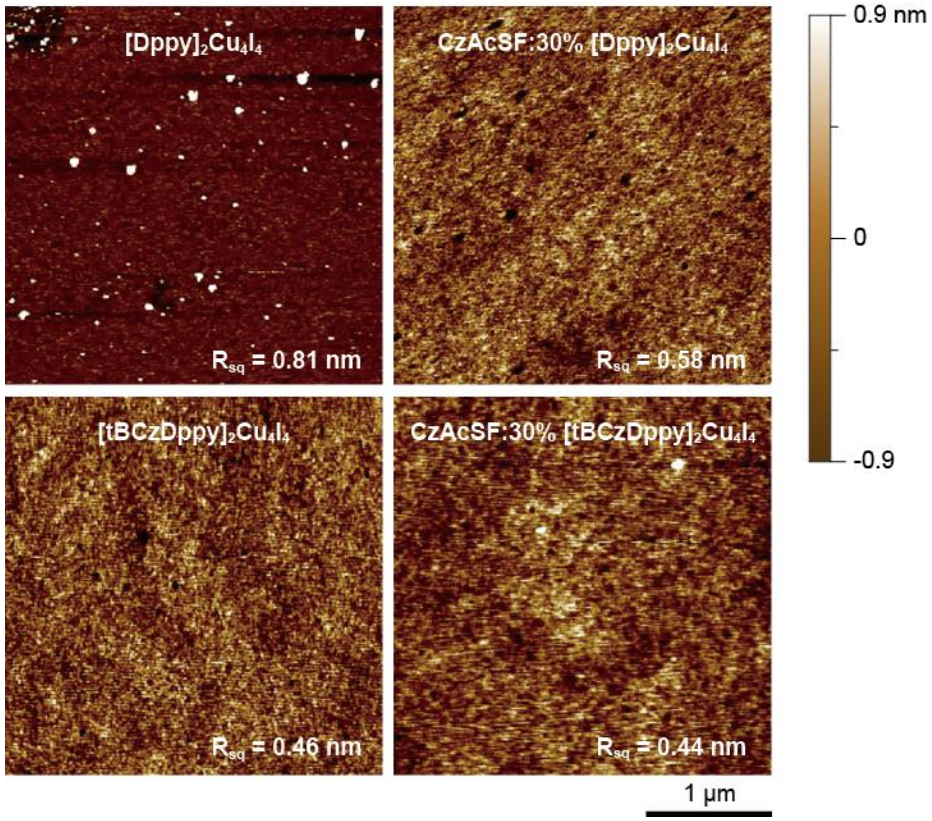

Supplementary Fig. 25 | AFM images of spin-coated films for neat clusters and CzAcSF:30% clusters with the thickness of 40 nm (testing area: 3 *μ*m × 3 *μ*m). R_{sq} refers to root-mean-square (RMS) roughness. Compared to $[Dppy]_2Cu_4I_4$ based films, RMS value of neat $[tBCzDppy]_2Cu_4I_4$ film is nearly halved, and RMS value of CzAcSF:$[tBCzDppy]_2Cu_4I_4$ is also reduced by one quarter, which demonstrate tBCz modification improves film formability, morphological stability and compatibility with organic host matrixes of

$[tBCzDppy]_2Cu_4I_4$.

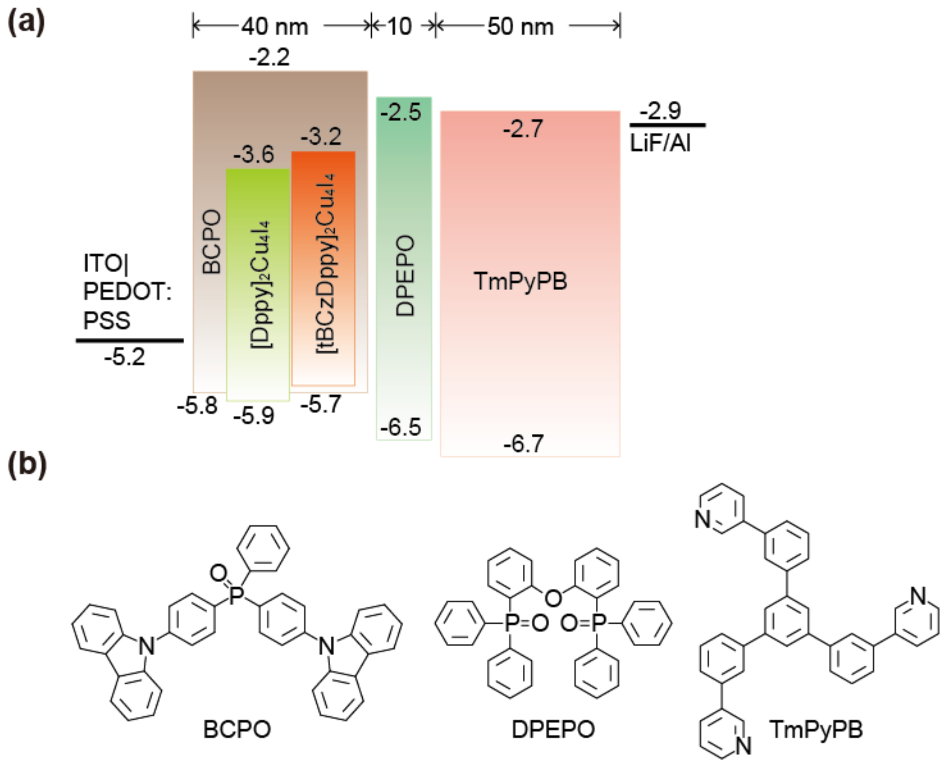

Supplementary Fig. 26 | EL performance of Cu_4I_4 clusters. (a) Device structure of ITO|PEDOT:PSS (40 nm)|BCPO:*x*% clusters (40 nm)|DPEPO (10 nm)|TmPyPB (50 nm)|LiF (1 nm)|Al (100 nm). (b) Chemical structures of employed materials.

2. Electroluminescence Properties

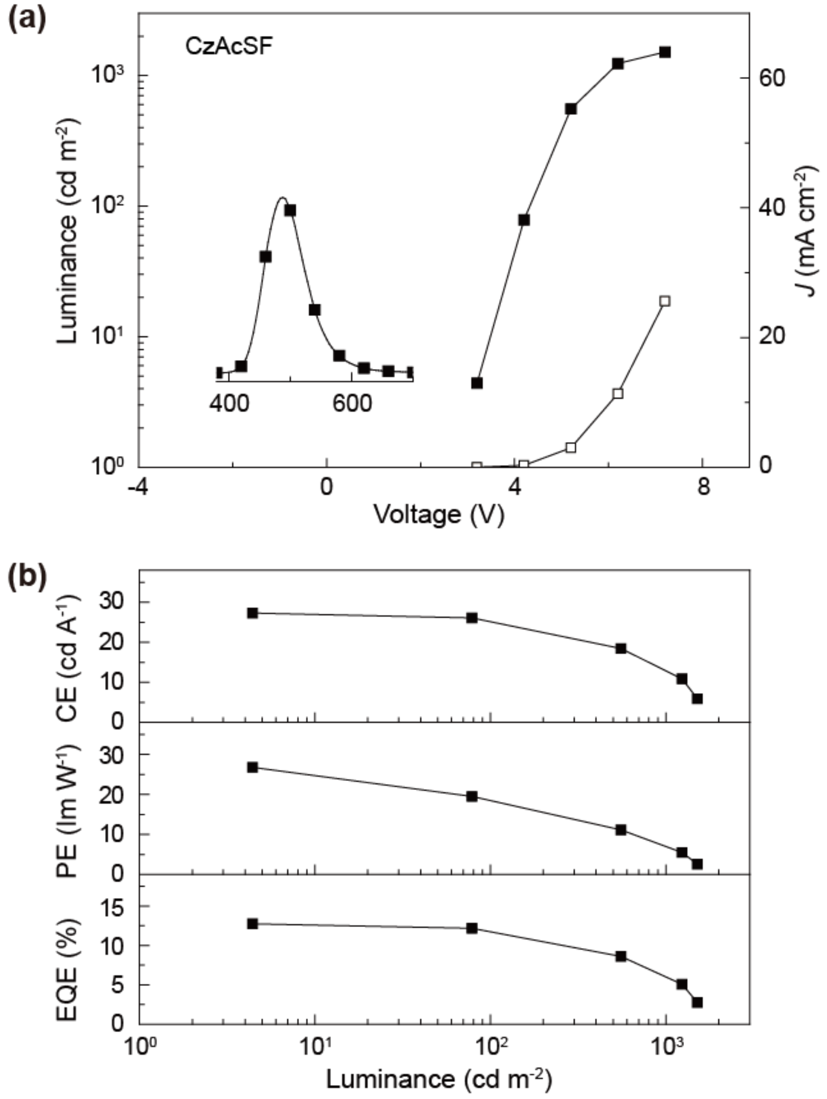

Supplementary Fig. 27 | EL performance of blue-emitting devices based on neat CzAcSF as EML. (a) EL spectra (inset) and Current density (J)-Voltage-Luminance characteristics. (b) Efficiencies vs. Luminance relationships.

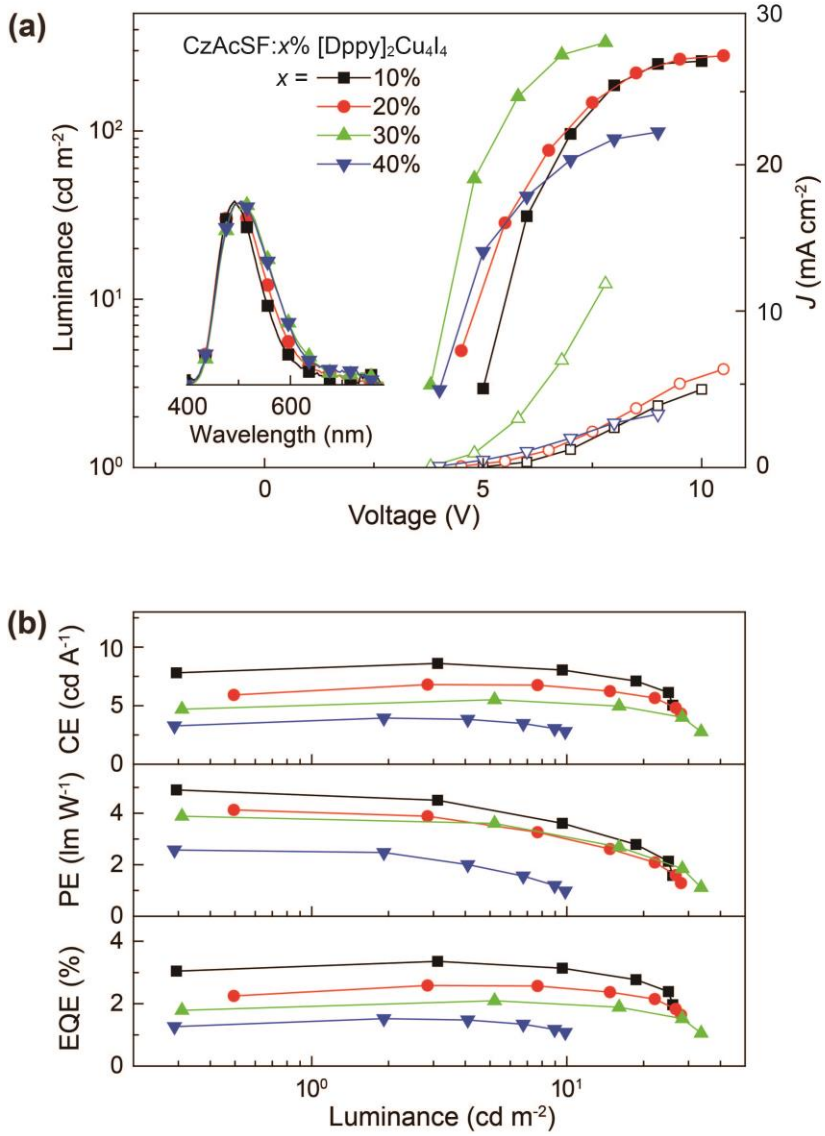

Supplementary Fig. 28 | EL performance of CzAcSF:*x*% $[Dppy]_2Cu_4I_4$ based CLEDs in doping concentration *x*% range of 10%-40%. (a) EL spectra (inset) and Current density (J)-Voltage-Luminance characteristics. (b) Efficiencies vs. Luminance relationships.

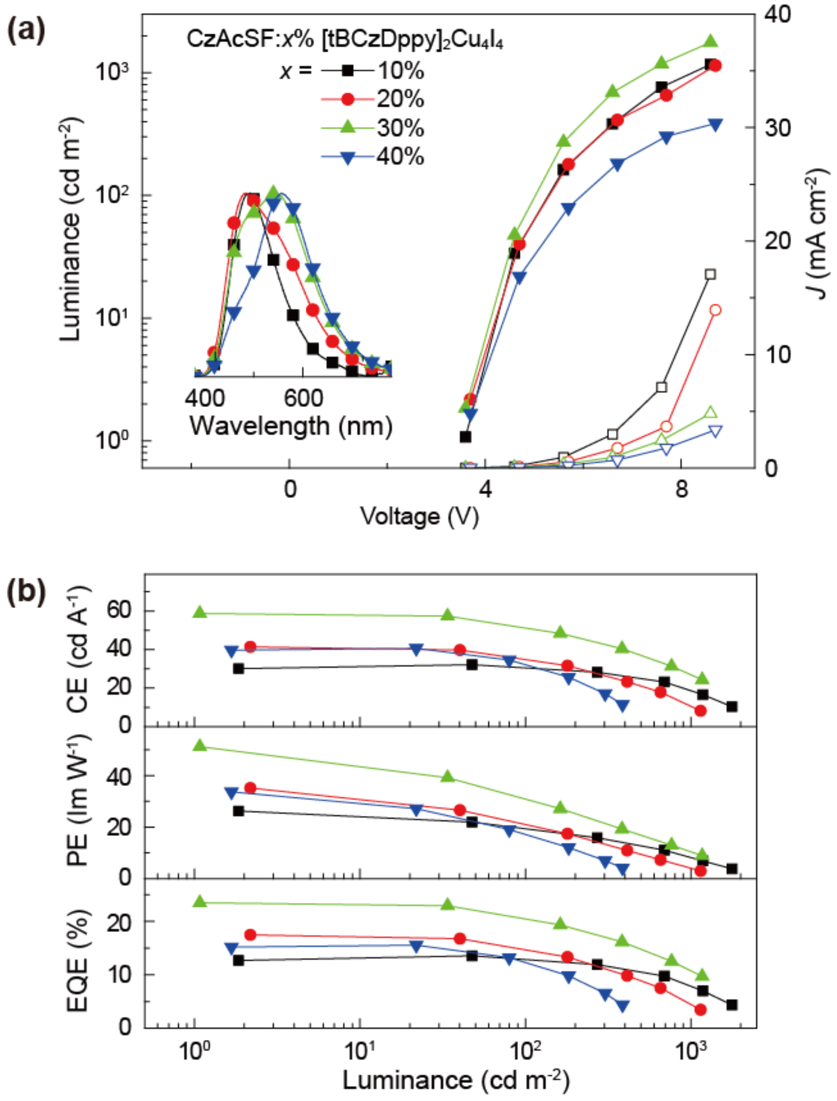

Supplementary Fig. 29 | EL performance of CzAcSF:*x*% $[tBCzDppy]_2Cu_4I_4$ based CLEDs in doping concentration *x*% range of 10%-40%. (a) EL spectra (inset) and Current density (J)-Voltage-Luminance characteristics. (b) Efficiencies vs. Luminance relationships.

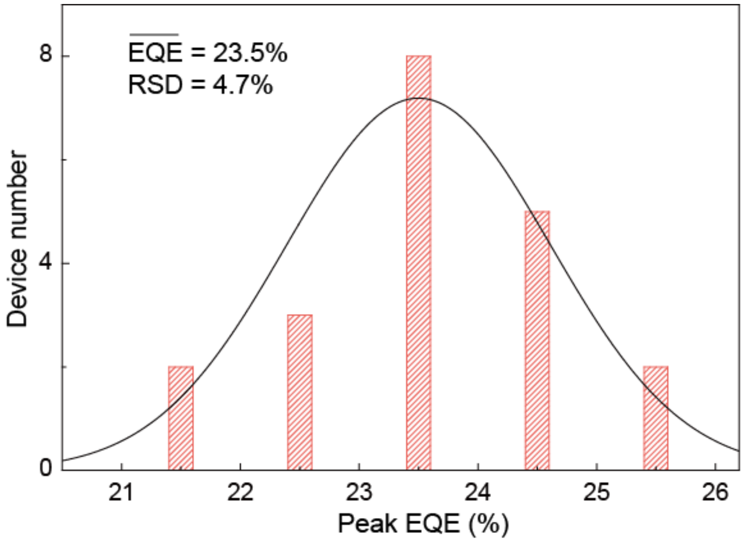

Supplementary Fig. 30 | Statistic analysis of the repeatability for the maximum EQE values of 20 CzAcSF:30% $[tBCzDppy]_2Cu_4I_4$ devices with the same structures. RSD refers relative standard deviation. The EQE variation followed normal distribution with a small RSD less than 5%.

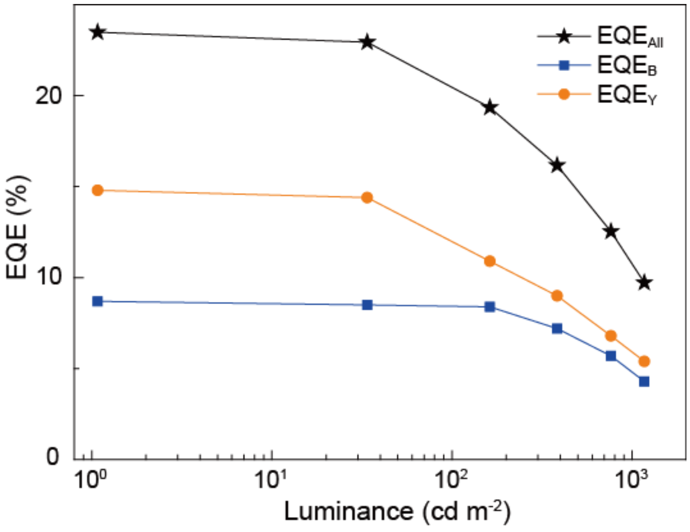

Supplementary Fig. 31 | EQE *vs.* Luminance relationships for whole white emission, and blue and yellow components for $\mathbf{[tBCzDppy]_2Cu_4I_4}$ based white CLEDs.

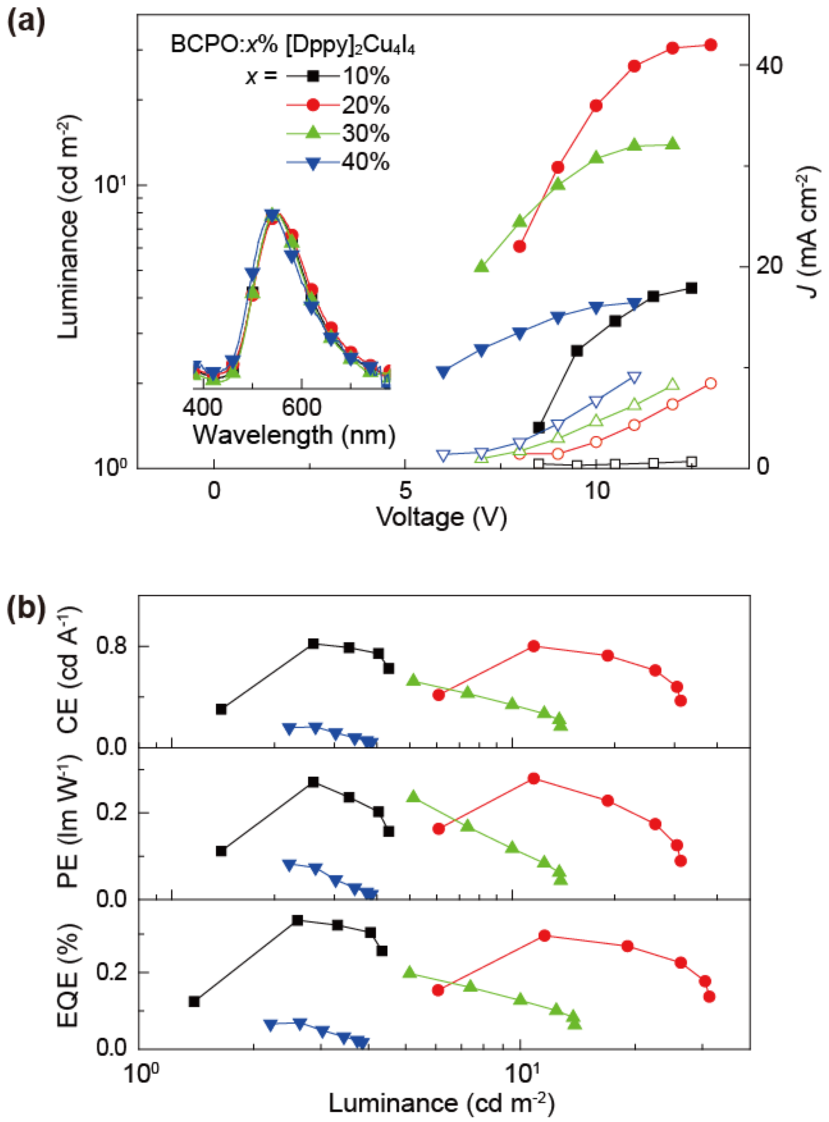

Supplementary Fig. 32 | EL performance of BCPO:*x*% $[Dppy]_2Cu_4I_4$ based CLEDs in doping concentration *x*% range of 10%-40%. (a) EL spectra (inset) and Current density (J)-Voltage-Luminance characteristics. (b) Efficiencies vs. Luminance relationships. Despite low luminance and efficiencies, the tendencies of the luminance and efficiencies for the devices were “first increase and then decrease”, corresponding to a turning point at $x = 20$.

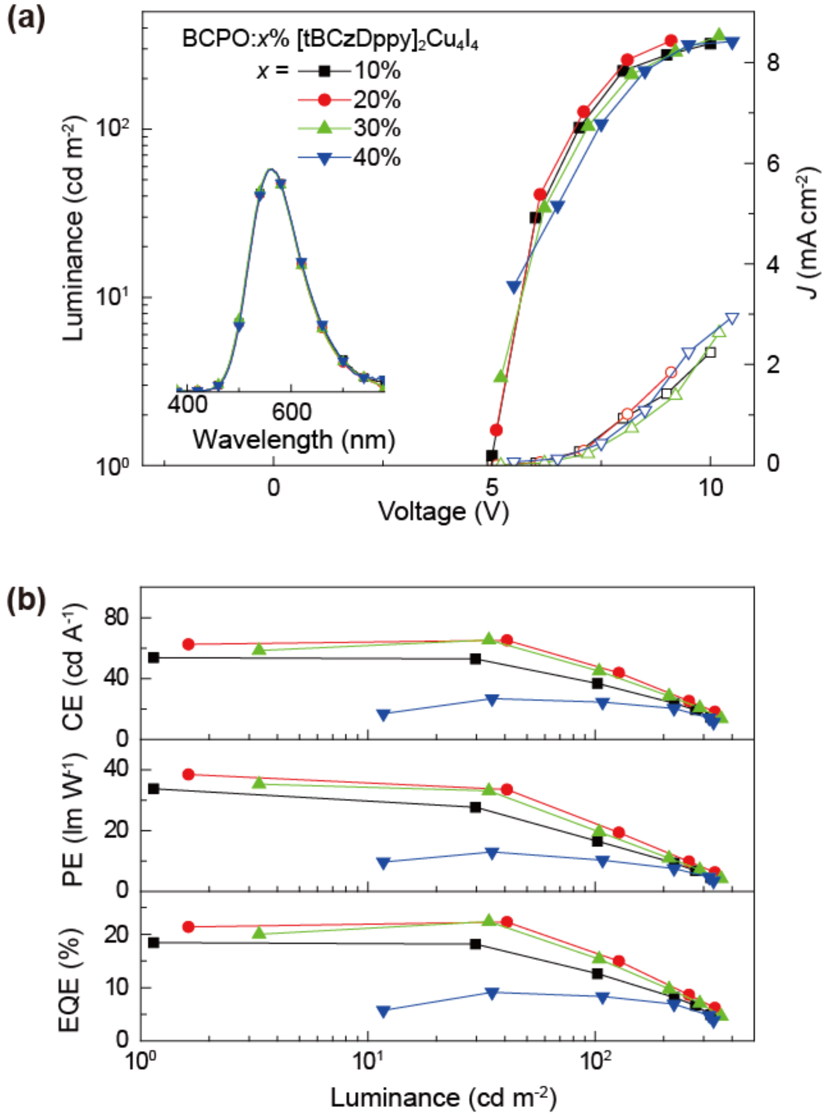

Supplementary Fig. 33 | EL performance of BCPO:*x*% $[tBCzDppy]_2Cu_4I_4$ based CLEDs in doping concentration *x*% range of 10%-40%. (a) EL spectra (inset) and Current density (J)-Voltage-Luminance characteristics. (b) Efficiencies vs. Luminance relationships.

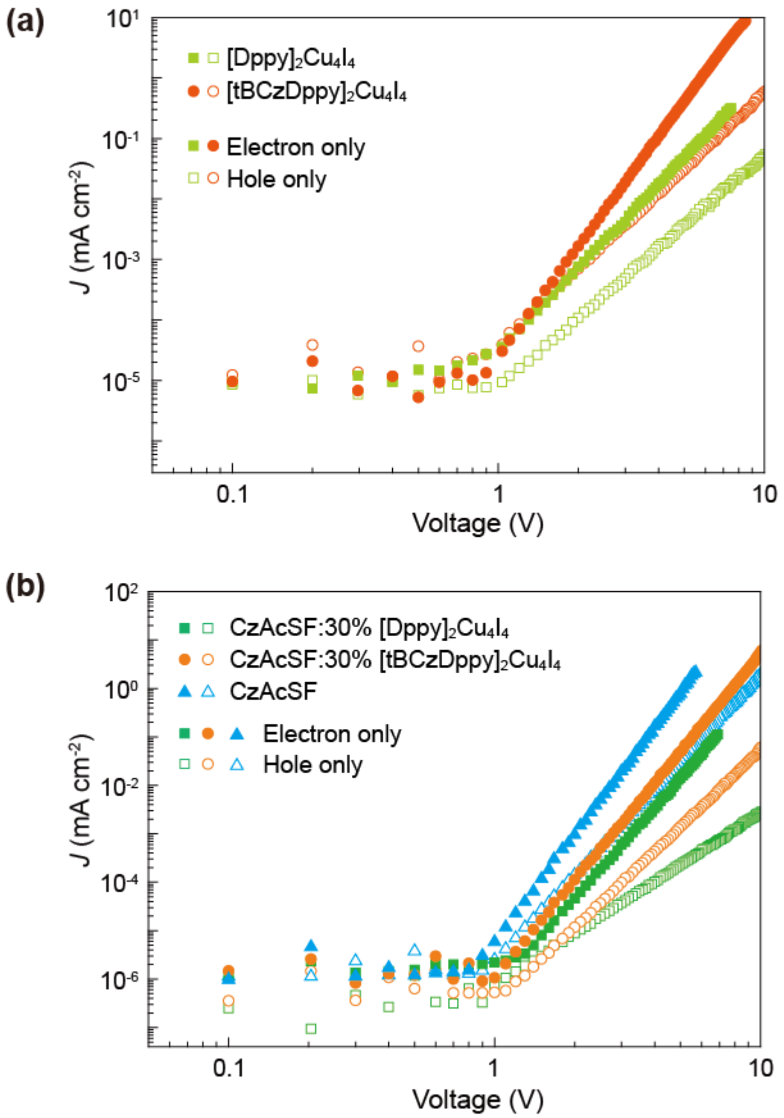

Supplementary Fig. 34 | *IV* characteristics of single-carrier-transporting devices based on $[Dppy]_2Cu_4I_4$ and $[tBCzDppy]_2Cu_4I_4$. (a) Voltage-current density (*J*) curves of single-carrier-transporting devices with configurations of ITO|PEDOT:PSS (40 nm)| $[Dppy]_2Cu_4I_4$ or $[tBCzDppy]_2Cu_4I_4$ (40 nm)|MoO_3 (6 nm)|Al (100 nm) for hole-only (shallow symbols) and ITO|LiF (1 nm)| $[Dppy]_2Cu_4I_4$ or $[tBCzDppy]_2Cu_4I_4$ (40 nm)|LiF (1 nm)|Al (100 nm) for electron-only (solid symbols), respectively. (b) Voltage-current density (*J*) curves of single-carrier-transporting devices for ITO|PEDOT:PSS (40 nm)|CzAcSF:*x*% $[Dppy]_2Cu_4I_4$ or $[tBCzDppy]_2Cu_4I_4$ (40 nm)|MoO_3 (6 nm)|Al (100 nm) and ITO|LiF (1 nm)|CzAcSF:*x*% $[Dppy]_2Cu_4I_4$ or $[tBCzDppy]_2Cu_4I_4$ (40 nm)|LiF (1 nm)|Al (100 nm), respectively. $x = 0$ for neat CzAcSF, and $x = 30\%$.

.

Supplementary Table 3. EL performance of CzAcSF:*x*% clusters based devices.

EML	*x* (wt%)	V[a] (V)	L_{max}[b] (cd m^{-2})	η[c]			λ_{EL} (nm) / CIE (x, y)[d]	CCT[e] (K)	CRI[f] (Ra)
				η_{CE} (cd A^{-1})	η_{PE} (lm W^{-1})	η_{EQE} (%)			
CzAcSF	0	3.2, 4.3, 5.9	1513	27.3, 25.0, 12.7	26.8, 18.5, 6.9	12.7, 11.8, 5.9	488/(0.16, 0.31)	-	-
CzAcSF:*x*% $[Dppy]_2Cu_4I_4$	10	5.0, 7.1, -	260	8.6, 8.0, -	4.9, 3.6, -	3.4, 3.1, -	492/(0.23, 0.40)	-	-
	20	4.5, 6.9, -	280	6.8, 6.5, -	4.1, 3.0, -	2.6, 2.5, -	496/(0.21, 0.38)	-	-
	30	3.8, 5.4, -	336	5.5, 5.2, -	3.9, 3.1, -	2.1, 2.0, -	500/(0.26, 0.43)	-	-
	40	4.0, 9, -	100	3.9, 2.8, -	2.6, 0.9, -	1.5, 1.1, -	504/(0.26, 0.42)	-	-
CzAcSF:*x*% $[tBCzDppy]_2Cu_4I_4$	10	3.6, 5.2, 8.2	1769	32.1, 30.0, 18.6	26.2, 19.1, 8.4	13.6, 12.8, 7.8	492/(0.23, 0.37)	-	-
	20	3.6, 5.3, 8.5	1145	41.4, 35.4, 10.6	35.1, 20.9, 4.0	17.5, 14.7, 4.4	484,555/(0.28, 0.37)	7632	72
	30	3.6, 5.0, 7.3	1169	58.7, 51.3, 27.1	51.2, 31.1, 10.5	23.5, 20.5, 10.8	475,544/(0.33, 0.41)	5675	81
	40	3.7, 6.0, -	386	40.6, 32.3, -	33.7, 16.9, -	15.6, 12.4, -	472,556/(0.38, 0.46)	4532	68

[a] Operation voltages for turn on, and at 100 and 1000 cd m^{-2}; [b] the maximum luminance; [c] EL efficiencies at the maximum, 100 and 1000 cd m^{-2}; [d] EL peak wavelengths and CIE coordinates at 1000 cd m^{-2}; [e] correlated color temperature; [f] color render index.

Supplementary Table 4. EL performance of BCPO:*x*% clusters based devices.

EML	*x* (wt%)	*V*[a] (V)	L_{max}[b] (cd m^{-2})	η[c] η_{CE} (cd A^{-1})	η_{PE} (lm W^{-1})	η_{EQE} (%)	λ_{EL} (nm) / CIE (x, y)[d]
BCPO:*x*% $[Dppy]_2Cu_4I_4$	10	8.5, -, -	4	0.8, -, -	0.3, -, -	0.3, -, -	548/(0.39, 0.51)
	20	6.1, -, -	31	0.8, -, -	0.3, -, -	0.3, -, -	556/(0.39, 0.50)
	30	7.0, -, -	14	0.5, -, -	0.2, -, -	0.2, -, -	544/(0.39, 0.52)
	40	6.0, -, -	4	0.2, -, -	0.1, -, -	0.1, -, -	540/(0.37, 0.50)
BCPO:*x*% $[tBCzDppy]_2Cu_4I_4$	10	5.0, 7.0, -	322	53.7, 37.3, -	33.7, 16.7, -	18.4, 12.7, -	560/(0.43, 0.52)
	20	3.5, 6.9, -	336	65.1, 48.5, -	38.5, 22.3, -	22.2, 16.5, -	564/(0.43, 0.52)
	30	3.3, 7.2, -	358	65.3, 45.8, -	35.3, 20.2, -	22.3, 15.6, -	560/(0.43, 0.52)
	40	3.1, 7.4, -	331	26.8, 24.5, -	13.0, 10.6, -	9.1, 8.5, -	564/(0.43, 0.52)

[a] Operation voltages for turn on, and at 100 and 1000 cd m^{-2}; [b] the maximum luminance; [c] EL efficiencies at the maximum, 100 and 1000 cd m^{-2}; [d] EL peak wavelengths and CIE coordinates at 1000 cd m^{-2}.

Supplementary Table 5. Comparison on EL performances of representative singly doped WOLEDs.

Fabrication method	Device structure	White or yellow emitter	V_{on}[a]	L_{max}[b] (cd m^{-2})	η[c]			CIE (x, y)	□$_{EL}$[d] (nm)	Ref.
					η_{CE} (cd A^{-1})	η_{PE} (lm W^{-1})	η_{EQE} (%)			
Vacuum evaporation	Blue Fluorescence + Yellow Fluorescence									
	ITO|NPB (40 nm)|Bepp2:**DCM** (0.5 wt%, 45 nm)|LiF (1 nm)|Al	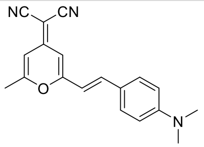	--	180	14.0	9.2	5.6	(0.33, 0.33)	450, 570	5
	ITO|NPB (40 nm)|DPVSBF:**DCJTB** (0.5 wt%, 11 nm)|Alq$_3$ (30 nm)|LiF (1 nm)|Al	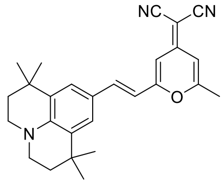	4.7	66000	8.0	5.35	3.3	(0.32, 0.34)	--	6
	ITO|MoO$_3$ (2 nm)|NPB (50 nm)|Spiro-Pye:**DCJTB** (5 wt%, 40 nm)|Bphen (30 nm)|LiF (1 nm)|Al	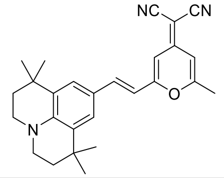	--	21070	18.1	8.8	8.2	(0.32, 0.31)	--	7
	Blue Fluorescence + Yellow Phosphorescence									
	ITO|NPB (40 nm)| 1:**(piq)$_2$Ir(acac)** (0.1 wt%, 20 nm)|BCP (10 nm)|Alq$_3$ (30 nm)|LiF (1 nm)|Al	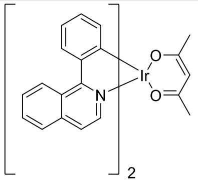	5.3	1200	7.9	4.7	5.2	(0.39, 0.31)	--	8

ITO|NPB (30 nm)|$Bepp_2$: **$(bzq)_2$Ir(dipba)** (2 wt%, 20 nm)|$Bepp_2$ (35 nm)|LiF (0.5 nm)|Al	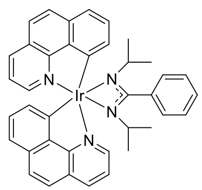	2.7	--	60.8	48.8	27.8	(0.35, 0.33)	--	9
ITO|NPB (30 nm)|TCTA (10 nm)|DADBT:**Ir(2-phq)$_3$** (0.1 wt%, 30 nm)|TPBI (30 nm)|LiF (1.5 nm)|Al	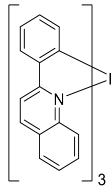	2.4	--	53.5	67.2	26.6	(0.46, 0.44)	--	10
ITO|CuPc (10 nm)|a-NPB (50 nm)|DPVBi:**4a** (0.2 wt%, 50 nm)|Alq_3 (10 nm)|LiF (1.2 nm)|Al	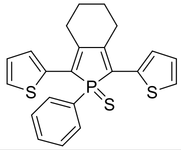	5.2	--	7.0	2.3	2.7	(0.31, 0.39)	444, 548	11
Blue TADF + Yellow TADF									
ITO|MoO_3 (6 nm)|mCP (80 nm)| *p*tBCzPO$_2$TPTZ:**4CzTPNBu** (1.5 wt%, 20 nm)|*p*TPOTPTZ (40 nm)|LiF (1 nm)|Al	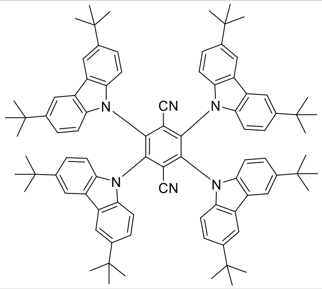	3.1	37160	52.7	55.1	23.6	(0.34, 0.36)	476, 572	12
Blue TADF + Yellow Fluorescence									
ITO|TAPC:20% MoO_3|TAPC|*m*CP|DMAC-DPS:**TBRb** (0.2 wt%)|DPEPO|BmPyPB:3% Li_2CO_3|Li_2CO_3|Al	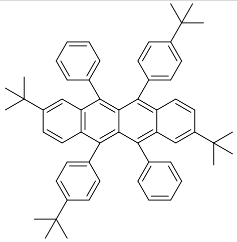	2.7	--	38.1	44.3	11.9	(0.38,0.49)	--	13

ITO|MoO_3 (6 nm)|NPB (70 nm)|mCP (5 nm)|Spiro-F:**4CzPNPh**(0.5 wt%, 20 nm,)|Spiro-F (5 nm)|TPBI (30 nm)|LiF (1 nm)|Al	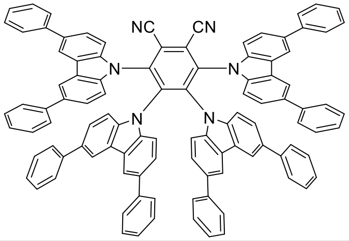	3.5	--	3.6	1.4	1.8	(0.29, 0.33)	--	14
Blue TADF + Yellow Phosphorescence									
ITO|HATCN (5 nm)|TAPC (50 nm)|TcTa (5 nm)|mCP-BP-DMAC:**Ir(MDQ)$_2$acac** (0.3 wt%, 20 nm)|TmPyPB (30 nm)|LiF (1 nm)|Al	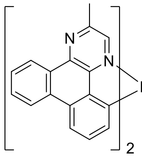	2.4	47320	45.7	50.5	20.6	(0.46, 0.43)	--	15
Single-Molecular White Emitters									
ITO|HATCN (10 nm)|NPD (40 nm)|TAPC (10 nm)|26mCPy:**Pd3O3** (5 wt%, 25 nm)|DPPS (10 nm)|BmPyPB (40 nm)|LiF|Al	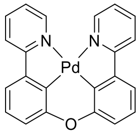	--	--	--	65.3	23.9	(0.34, 0.47)	--	16
ITO|HATCN (10 nm)|NPD (40 nm)|TrisPCz (10 nm)| mCBP: **Pt2O2p2m** (20 wt%, 20 nm)|BAlq (10 nm)|Bpytp (40 nm)|Liq (2 nm)|Al	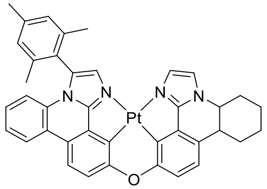	--	4432	--	--	13.6	(0.39, 0.46)	--	17
ITO|HATCN (10 nm)|NPD (40 nm)|TAPC (10 nm)|26mCPy:**Pt2O2** (16 wt%, 25 nm)|DPPS (10 nm) |BmPyPB (40 nm)|LiF|Al	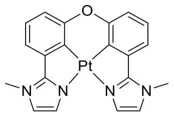	--	--	59.0	46.1	24.6	(0.48, 0.48)	--	18

Spin coating	**Blue Fluorescence + Yellow Fluorescence**									
	ITO|CH 8000 (40 nm)|PVK (30 nm)|G0:**CN-DPASDB** (0.15 wt%, 50 nm)|Ba (4 nm)|Al	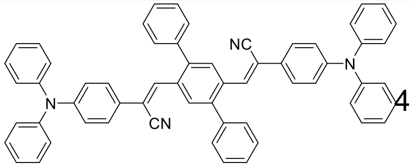	4.0	29700	7.8	4.6	3.0	(0.26, 0.28)	--	[19]
	**Blue Fluorescence + Yellow Phosphorescence**									
	ITO|PEDOT:PSS (40 nm)|PTPATPPO: **Ir(2-phq)$_2$acac** (2 wt%, 60 nm)|SPPO13 (70 nm)|Al	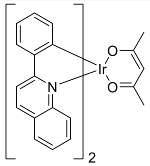	4.9	11962	10.5	7.2	6.1	(0.40, 0.34)	--	[20]
	ITO|PEDOT:PSS (30 nm)|DTAF (25 nm)|CphBzIm:**(pbi)$_2$Ir(acac)** (0.1 wt%, 25 nm)|TPBI (50 nm)|LiF (0.5 nm)|Al	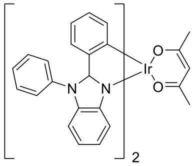	2.5	26500	15.5	12.8	7.0	(0.31, 0.33)	--	[21]
	**Blue Phosphorescence + Yellow Phosphorescence**									
	ITO|PEDOT:PSS (40 nm)|B-G2: **Ir(Flpy-CF$_3$)$_3$** (0.3 wt%, 30 nm)|TPCz (8 nm)|TmPyPb (42 nm)|LiF (1 nm)|Al	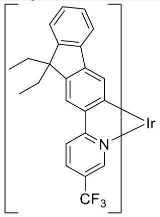	2.9	--	46.0	47.4	16.3	(0.33, 0.41)	--	[22]
	ITO|PEDOT:PSS|PF8:**IrDBQ** (0.5 wt%) |Ba (30 nm)|Al	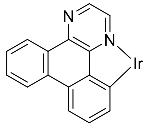	4.3	170	--	0.31	2.3	(0.32, 0.23)	--	[23]

ITO|PEDOT:PSS-PFI (30 nm)|SDPS-4PhCz:**TXO-TPA** (0.2 wt%, 60 nm)|TMPYPB (65 nm)|LiF (1 nm)|Al	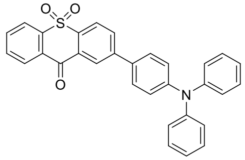	3.1	3000	24.1	22.9	10.4	(0.35, 0.40)	--	24
ITO|PEDOT:PSS (45 nm)|TAPC (20 nm)|PyDCN–DMAC:**PP–PXZ** (0.3 wt%, 20 nm)|TmPyPB (40 nm)|LiF (1 nm)|Al	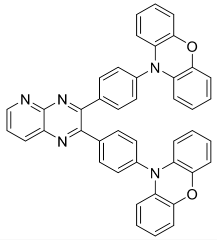	2.6	9000	44.8	49.0	18.5	(0.38, 0.44)	472, 581	25
Blue TADF + Yellow Phosphorescence									
ITO|PEDOT:PSS (40 nm)|CzAcSF: $[tBCzDppy]_2Cu_4I_4$ (30 wt%, 40 nm)|DPEPO (10 nm)|TmPyPB (50 nm)|LiF (1 nm)|Al	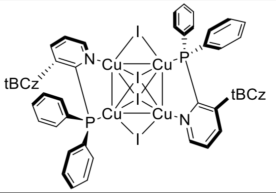	3.6	1169	58.7	51.2	23.5	(0.33, 0.41)	475, 544	This work

[a] Turn-on voltages; [b] the maximum luminance; [c] the maximum efficiencies; [d] EL peak wavelengths.

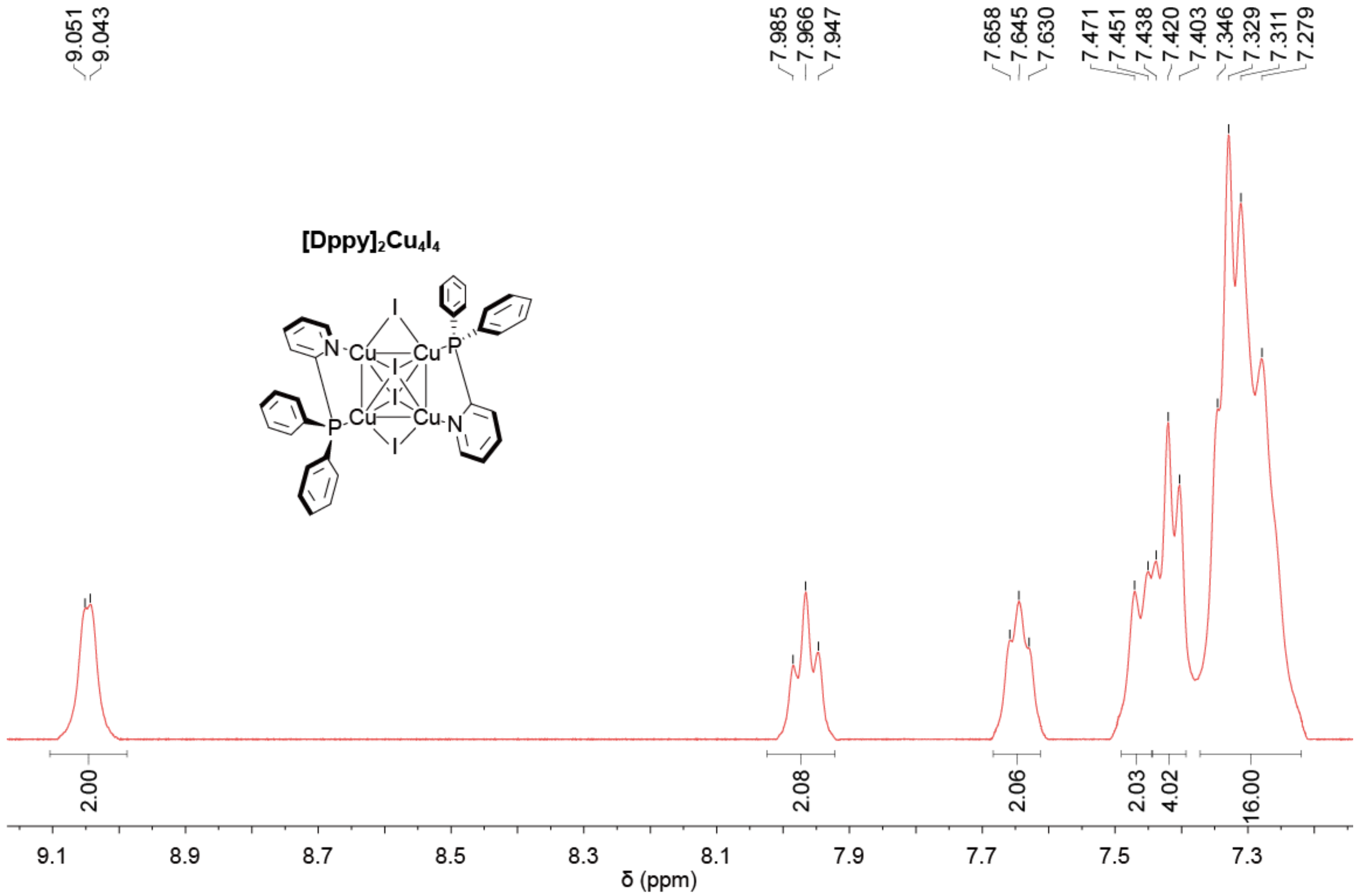

Supplementary Fig. 35 | ^{1}H NMR spectrum of $[Dppy]_2Cu_4I_4$ (TMS, $CDCl_3$, 400 MHz).

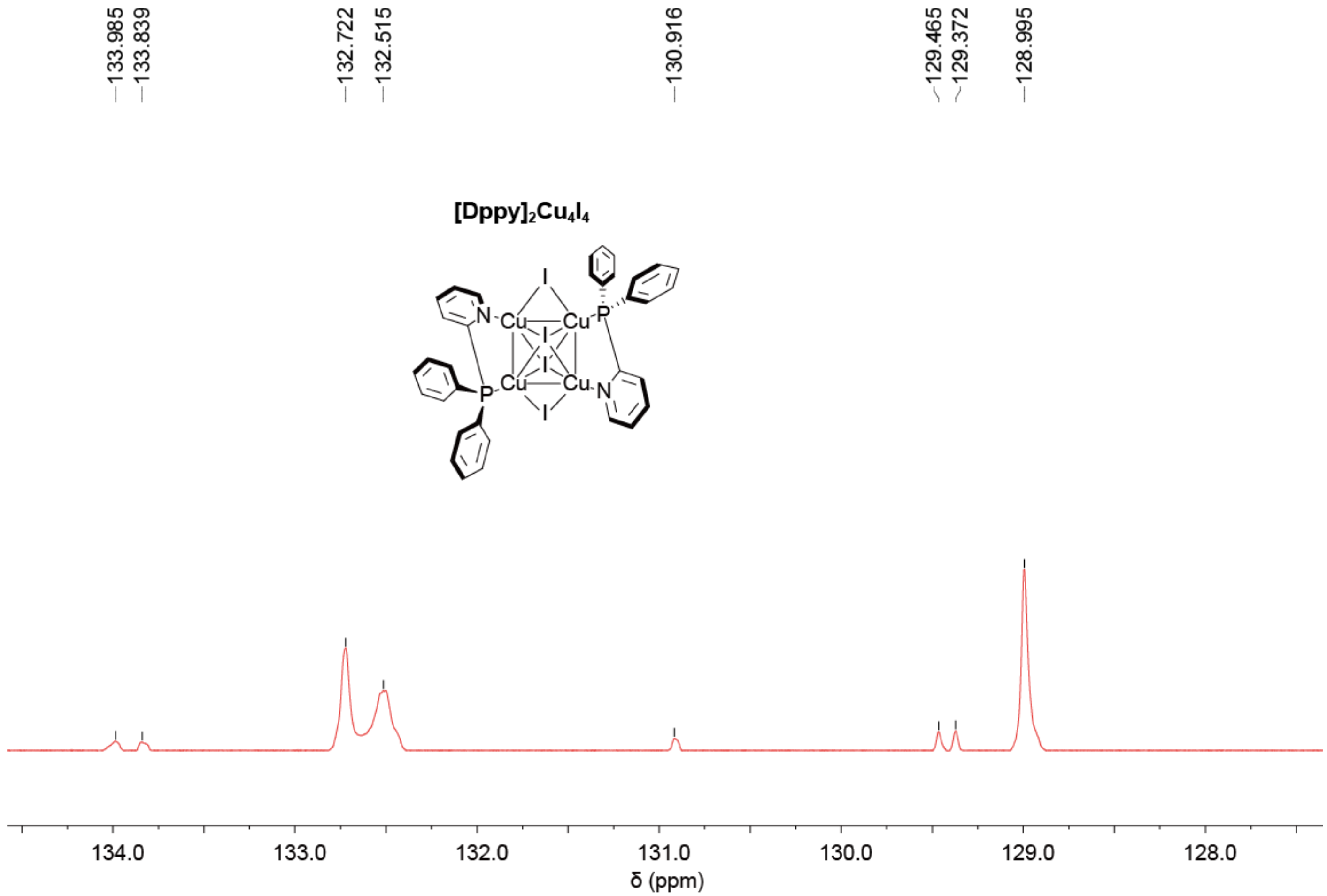

Supplementary Fig. 36 | ^{13}C NMR spectrum of $[Dppy]_2Cu_4I_4$ (TMS, $CDCl_3$, 100 MHz).

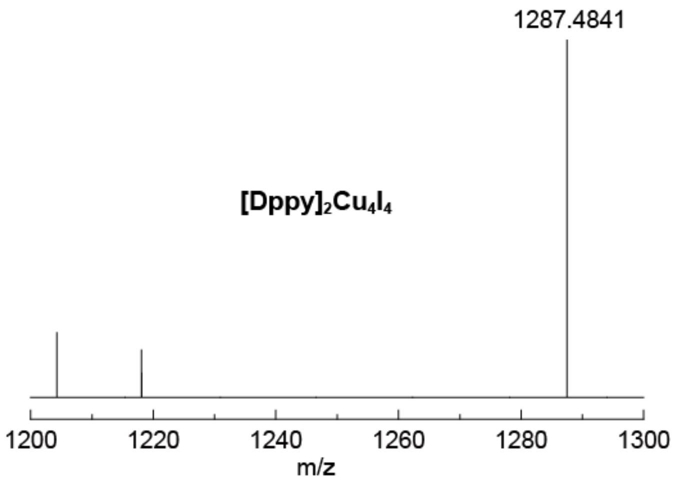

Supplementary Fig. 37 | ESI-MS spectrum of $[Dppy]_2Cu_4I_4$.

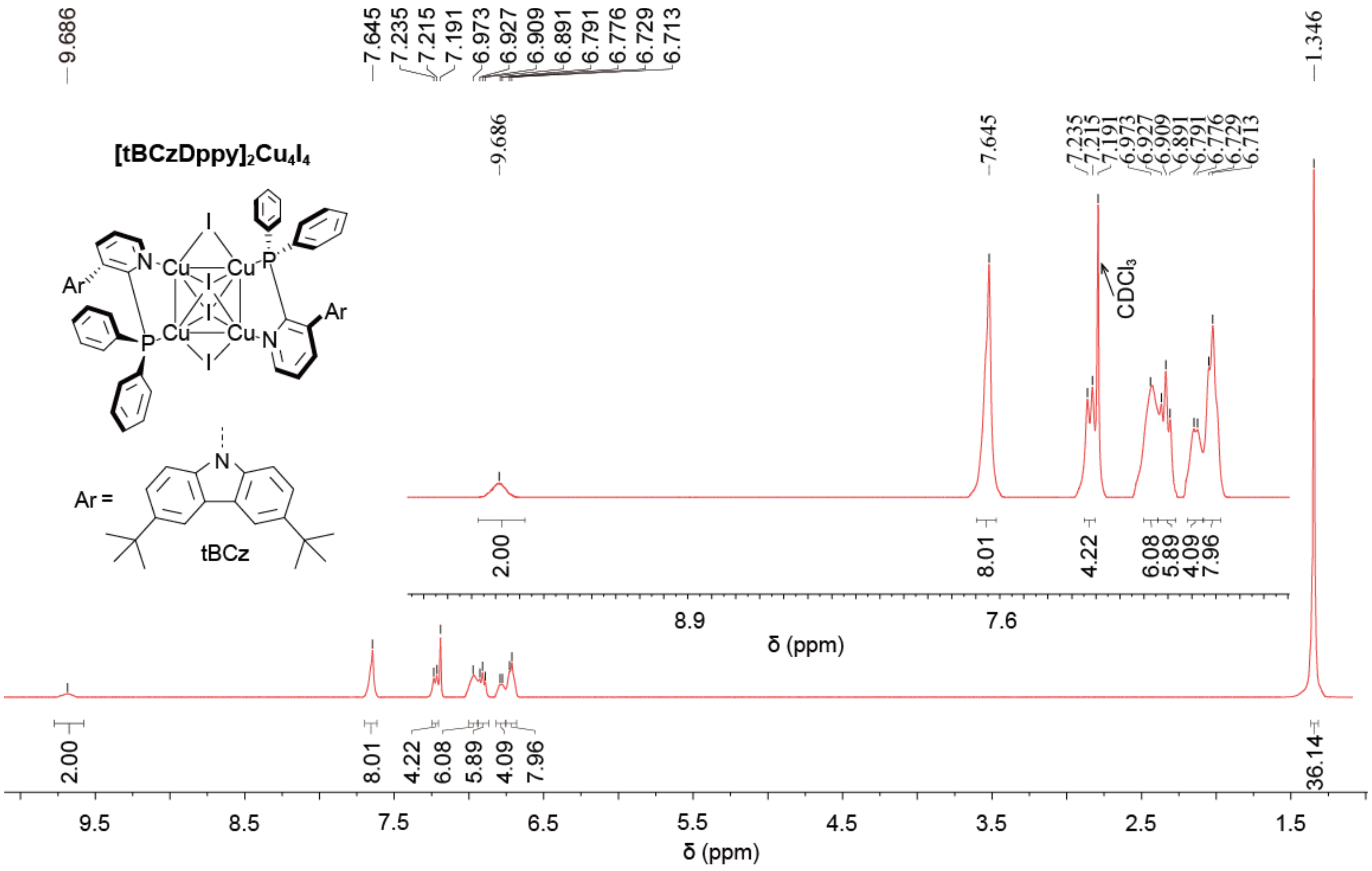

Supplementary Fig. 38 | ^{1}H NMR spectrum of $[tBCzDppy]_2Cu_4I_4$ (TMS, $CDCl_3$, 400 MHz).

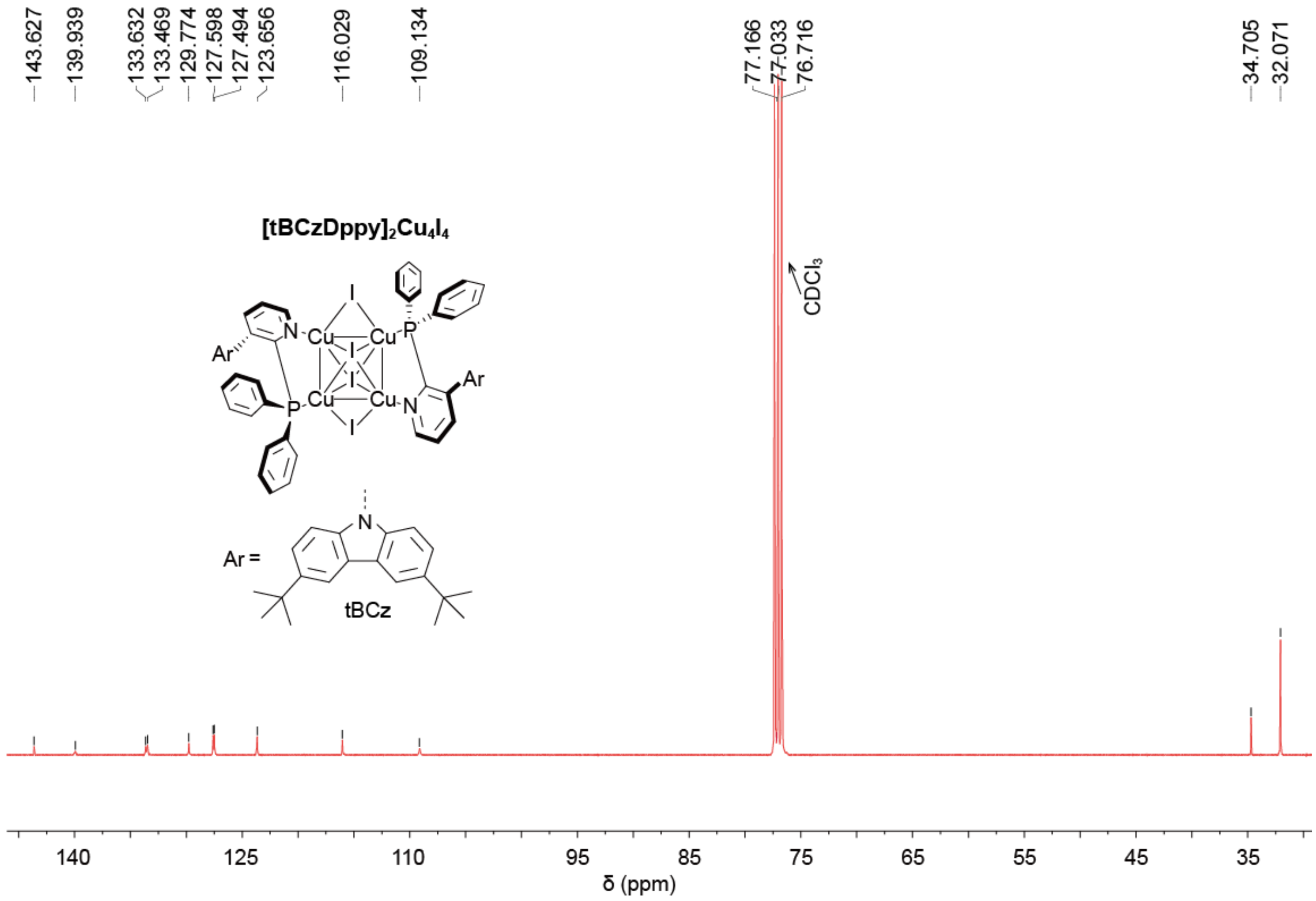

Supplementary Fig. 39 | ^{13}C NMR spectrum of [tBCzDppy]$_2$Cu$_4$I$_4$ (TMS, CDCl$_3$, 100 MHz).

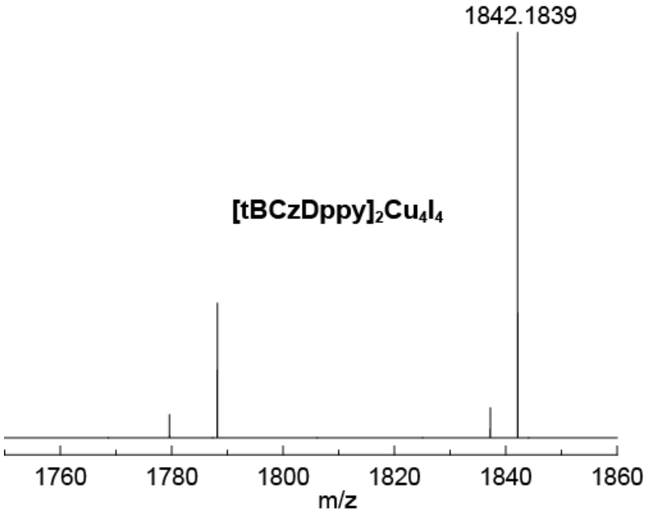

Supplementary Fig. 40 | ESI-MS spectrum of $[tBCzDppy]_2Cu_4I_4$.

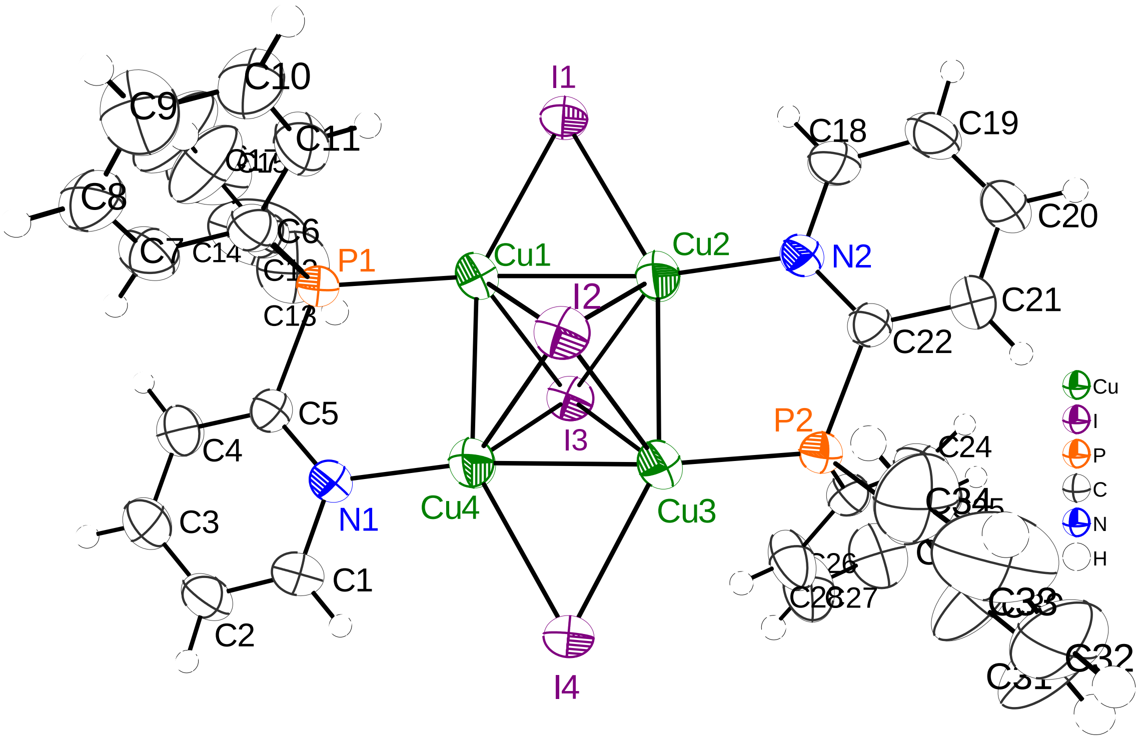

Supplementary Fig. 41 | ORTEP diagram of $[Dppy]_2Cu_4I_4$ with 50% probability ellipsoids.

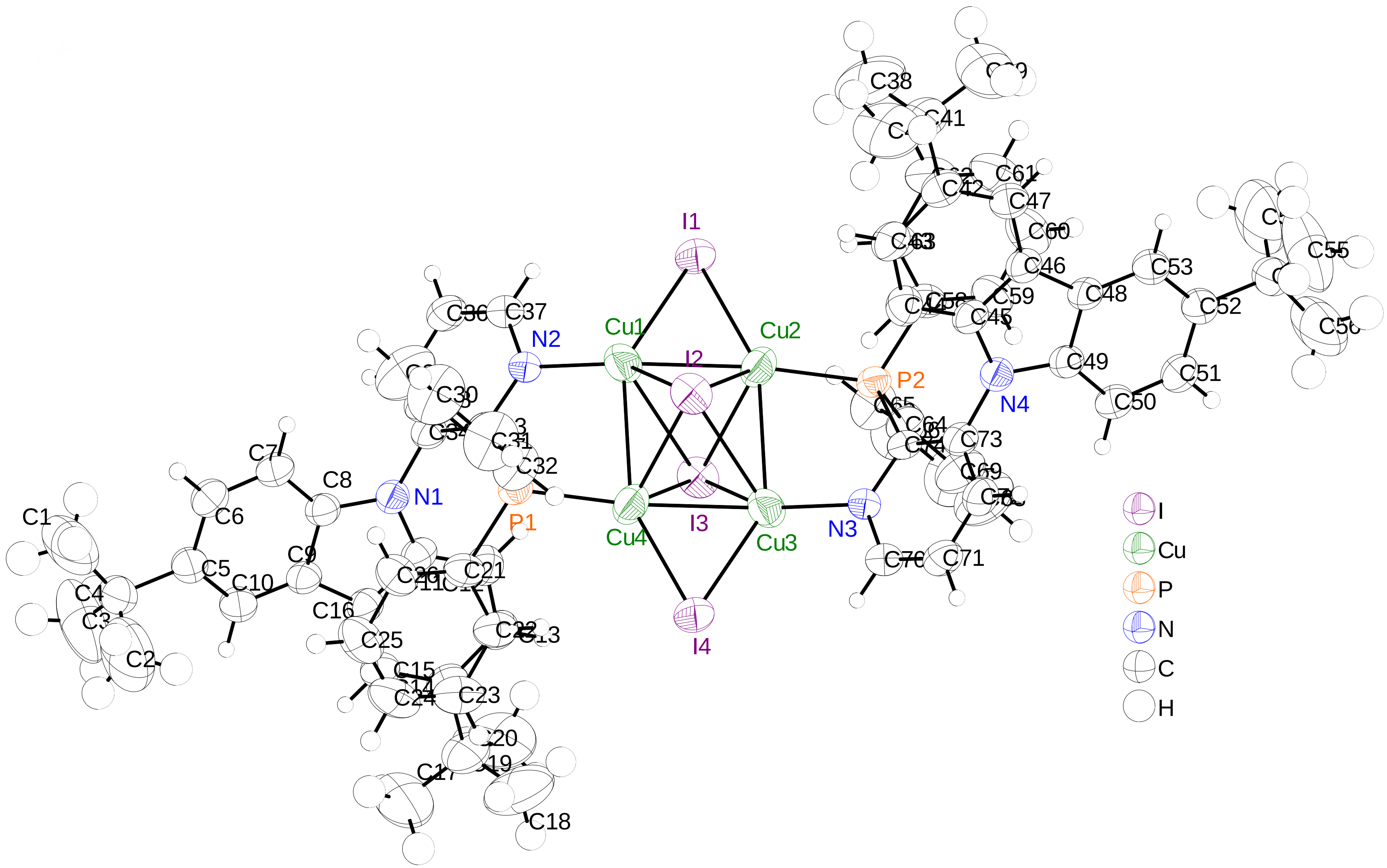

Supplementary Fig. 42 | ORTEP diagram of $[tBCzDppy]_2Cu_4I_4$ with 50% probability ellipsoids.

Supplementary References

[1] Xie, M., Han, C., Zhang, J., Xie, G. & Xu, H. White Electroluminescent Phosphine-Chelated Copper Iodide Nanoclusters. *Chem. Mater.* **29**, 6606-6610 (2017).

[2] Becke, A. D. Density-functional thermochemistry. III. The role of exact exchange. *J. Chem. Phys.* **98**, 5648-5652 (1993).

[3] Lee, C., Yang, W. & Parr, R. G. Development of the Colle-Salvetti correlation-energy formula into a functional of the electron density. *Phys. Rev. B* **37**, 785-789 (1988).

[4] Gaussian 09 v. D. 1 (Gaussian, Inc., Wallingford CT, USA, 2009).

[5] Yang, Y. et al. High-efficiency and high-quality white organic light-emitting diode employing fluorescent emitters. *Org. Electron.* **12**, 29-33 (2011).

[6] Chuen, C. H., Tao, Y. T., Wu, F. I. & Shu, C. F. White organic light-emitting diodes based on 2,7-bis(2,2-diphenylvinyl)-9,9′-spirobifluorene: Improvement in operational lifetime. *Appl. Phys. Lett.* **85**, 4609-4611 (2004).

[7] Yang, S.-H. & Huang, T.-L. High fluorescence efficiency of dual-wavelength white OLED with NPB emission and triplet annihilation. *Opt. Mater.* **111**, 110725 (2021).

[8] Tao, Y. et al. Multifunctional bipolar triphenylamine/oxadiazole derivatives: highly efficient blue fluorescence, red phosphorescence host and two-color based white OLEDs. *Chem. Commun.* **1**, 77-79 (2009).

[9] Peng, T. et al. Highly efficient white organic electroluminescence device based on a phosphorescent orange material doped in a blue host emitter. *J. Mater. Chem.* **21**, 3551-3553 (2011).

[10] Ye, J. et al. Management of Singlet and Triplet Excitons in a Single Emission Layer: A Simple Approach for a High-Efficiency Fluorescence/Phosphorescence Hybrid White Organic Light-Emitting Device. *Adv. Mater.* **24**, 3410-3414 (2012).

[11] Fadhel, O. et al. Tunable Organophosphorus Dopants for Bright White Organic Light-Emitting Diodes with Simple Structures. *Adv. Mater.* **21**, 1261-1265 (2009).

[12] Ding, D. et al. Highly Efficient and Color-Stable Thermally Activated Delayed Fluorescence White Light-Emitting Diodes Featured with Single-Doped Single Emissive Layers. *Adv. Mater.* **32**, 1906950 (2020).

[13] Wu, Z. et al. Management of Singlet and Triplet Excitons: A Universal Approach to High-Efficiency All Fluorescent WOLEDs with Reduced Efficiency Roll-Off Using a Conventional Fluorescent Emitter. *Adv. Opt. Mater.* **4**, 1067-1074 (2016).

[14] Xia, D. et al. Oligofluorene with multiple spiro-connections: its and their use in blue and white OLEDs. *New J. Chem.* **43**, 3788-3792 (2019).

[15] Wu, X. et al. Robust sky-blue aggregation-induced delayed fluorescence materials for high-performance top-emitting OLEDs and single emissive layer white OLEDs. *Chem. Engin. J.* **451**, 138919 (2023).

[16] Fleetham, T. et al. Efficient and stable single-doped white OLEDs using a palladium-based phosphorescent excimer. *Chem. Sci.* **8**, 7983-7990 (2017).

[17] Wu, J., Ameri, L., Cao, L. & Li, J. Efficient excimer-based white OLEDs with reduced efficiency roll-off. *Appl. Phys. Lett.* **118**, 073301 (2021).

[18] Fleetham, T., Huang, L. & Li, J. Tetradentate Platinum Complexes for Efficient and Stable Excimer-Based White OLEDs. *Adv. Funct. Mater.* **24**, 6066-6073 (2014).

[19] Jiang, Z. et al. Highly Efficient, Solution Processed Electrofluorescent Small Molecule White Organic Light-Emitting Diodes with a Hybrid Electron Injection Layer. *ACS Appl. Mater. Interfaces* **6**, 8345-8352 (2014).

[20] Wang, J. et al. A novel blue fluorescent polymer for solution-processed fluorescent–phosphorescent hybrid WOLEDs. *J. Mater. Chem. C* **3**, 2856-2864 (2015).

[21] Hung, W.-Y. et al. A new benzimidazole/carbazole hybrid bipolar material for highly efficient deep-blue electrofluorescence, yellow–green electrophosphorescence, and two-color-based white OLEDs. *J. Mater. Chem.* **20**, 10113-10119 (2010).

[22] Wang, S. et al. Improving the Power Efficiency of Solution-Processed Phosphorescent WOLEDs with

a Self-Host Blue Iridium Dendrimer. *Adv. Opt. Mater.* **5**, 1700514 (2017).
[23] Gutiérrez–Llorente, A. Excimer emission of Ir complex for solution processed single emitting layer white OLEDs. *Org. Electron.* **63**, 305-309 (2018).
[24] Wang, R. et al. Solution-processed white organic light-emitting diodes with bi-component emitting layer based on symmetry blue spiro-sulfone derivative. *Org. Electron.* **71**, 24-30 (2019).
[25] Dong, R. et al. Acceptor modulation for blue and yellow TADF materials and fabrication of all-TADF white OLED. *Mater. Chem. Front.* **6**, 40-51 (2022).
